# Framework of the *Alu* Subfamily Evolution in the Platyrrhine Three-Family Clade of Cebidae, Callithrichidae, and Aotidae

**DOI:** 10.3390/genes14020249

**Published:** 2023-01-18

**Authors:** Jessica M. Storer, Jerilyn A. Walker, Jasmine N. Baker, Shifat Hossain, Christian Roos, Travis J. Wheeler, Mark A. Batzer

**Affiliations:** 1 Department of Biological Sciences, Louisiana State University, 202 Life Sciences Building, Baton Rouge, LA 70803, USA; jessica.storer@isbscience.org (J.M.S.); jawalker@lsu.edu (J.A.W.); 2 Institute for Systems Biology, Seattle, WA 98109, USA; 3 Department of Molecular and Human Genetics, Baylor College of Medicine, Houston, TX 77030, USA; jnb1@bcm.edu; 4 Department of Pharmacy Practice & Science, University of Arizona, Tucson, AZ 85721, USA; shifathossain@arizona.edu (S.H.); twheeler@arizona.edu (T.J.W.); 5 Gene Bank of Primates and Primate Genetics Laboratory, German Primate Center, Leibniz Institute for Primate Research, 37077 Göttingen, Germany; croos@dpz.eu

**Keywords:** Cebidae, Aotidae, Callithrichidae, Platyrrhini, *Alu*, evolution, retrotransposon, genomics, SCULU

## Abstract

The history of *Alu* retroposons has been choreographed by the systematic accumulation of inherited diagnostic nucleotide substitutions to form discrete subfamilies, each having a distinct nucleotide consensus sequence. The oldest subfamily, *Alu*J, gave rise to *Alu*S after the split between Strepsirrhini and what would become Catarrhini and Platyrrhini. The *Alu*S lineage gave rise to *Alu*Y in catarrhines and to *Alu*Ta in platyrrhines. Platyrrhine *Alu* subfamilies Ta7, Ta10, and Ta15 were assigned names based on a standardized nomenclature. However, with the subsequent intensification of whole genome sequencing (WGS), large scale analyses to characterize *Alu* subfamilies using the program COSEG identified entire lineages of subfamilies simultaneously. The first platyrrhine genome with WGS, the common marmoset *(Callithrix jacchus*; [caljac3]), resulted in *Alu* subfamily names sf0 to sf94 in an arbitrary order. Although easily resolved by alignment of the consensus sequences, this naming convention can become increasingly confusing as more genomes are independently analyzed. In this study, we reported *Alu* subfamily characterization for the platyrrhine three-family clade of Cebidae, Callithrichidae, and Aotidae. We investigated one species/genome from each recognized family of Callithrichidae and Aotidae and of both subfamilies (Cebinae and Saimiriinae) of the family Cebidae. Furthermore, we constructed a comprehensive network of *Alu* subfamily evolution within the three-family clade of platyrrhines to provide a working framework for future research. *Alu* expansion in the three-family clade has been dominated by *Alu*Ta15 and its derivatives.

## 1. Introduction

### 1.1. History of Alu Elements

The discovery in the mid-1970′s that a large fraction of the human genome is occupied by interspersed repetitive sequences, each about 300 nucleotides long [1], prompted scientists to investigate the architecture of this repeated DNA sequence by subjecting it to cleavage using a variety of different restriction enzymes [2]. Only one out of thirteen different restriction enzymes resulted in cutting the DNA into two fragments. That endonuclease was *Alu*I, and thus the phrase “*Alu* family of repeats” was coined [2]. The division of *Alu* sequences into subfamilies based on unique nucleotide substitutions began with the designation of *Alu*J (Jurka) and *Alu*S (Smith) [3], named after the researchers who discovered them, and was quickly followed by many others including, but not limited to, CS (catarrhine-specific), PS (primate-specific), HS (human-specific), and *Alu*Y (meaning young) [4,5,6]. The *Alu*S subfamily arose from *Alu*J after the split between Strepsirrhini and what would become Platyrrhini and Catarrhini [3,7]. The *Alu*Y lineage of subfamilies are only present in catarrhines [4,8,9], and thus only *Alu*J and *Alu*S families and their derivatives are present in platyrrhines.

In the mid-1990′s, a standardized nomenclature for *Alu* repeats was introduced [10] to bring together this rapidly emerging field of research that lacked formal guidelines. Using the new standard, lower case letters after the J, S, or Y indicate a new subfamily derived from that *Alu* lineage, followed by the number of diagnostic nucleotide changes from the parent subfamily. The HS-1 subfamily became Ya5 and HS-2 became Ya8 [10], providing an organized hierarchy associated with the naming convention. This new system also proposed that *Alu* variants identified in non-human primates be denoted with an abbreviated genus and species to indicate the taxon in which the *Alu* subfamily was discovered, such as Ya5*Ptr* for a variant of *Alu*Ya5 that was discovered in *Pan troglodytes* (common chimpanzee). This standardized nomenclature was generally followed over the next several years, introducing subfamily designations such as *Alu*MacYa3 discovered in *Macaca mulatta* (rhesus macaque) [11], *Alu*Yd3a1_gib, discovered in *Nomascus leucogenys* (northern white-cheeked gibbon) [12], *Alu*Ye5b5_*Pongo* discovered in orangutan [13], and a new *Alu*L lineage of subfamilies unique to Lemuridae (true lemurs) [14]. The first platyrrhine-specific *Alu* subfamily discovered was given the new name *Alu*T, because it was created by a fusion event between *Alu*Sc and *Alu*Sp elements [15]. This platyrrhine specific *Alu*Ta-lineage includes Ta7, Ta10, and Ta15, with *Alu*Ta15 thought to be limited to the three-family clade of Cebidae, Callithrichidae, and Aotidae platyrrhines (we follow here the new classification in which callithrichids, owl monkeys, squirrel monkeys, and capuchin monkeys are divided into three families, Callithrichidae, Aotidae, and Cebidae, as opposed to earlier nomenclature that placed these taxa together into a single family, Cebidae) [15,16].

### 1.2. Alu Analyses of Whole Genomes

In recent years, the explosion of whole genome sequencing (WGS) projects has produced high quality genome assemblies for many non-human primates. This has permitted genome wide analyses of repeat content using RepeatMasker, as well as *Alu* subfamily identification using the COSEG program based on co-segregating mutations [17]. This methodology has greatly accelerated the number of primate genomes characterized for the *Alu* element subfamily content [18,19,20,21,22]. However, the COSEG output assigns subfamily names arbitrarily, starting with subfamily “0” and going through to subfamily x, in no particular order and with no regard for the step-wise accumulation of diagnostic mutations. For example, the COSEG output for lineage-specific *Alu* subfamilies in *Papio* baboons [20] was reported as subfamily0 to subfamily128, for the rhesus macaque genome [Mmul_10] an *Alu* network was reported for subfamily 0 to subfamily 150 [21], and for bonobo, *Pan paniscus* [panPan3], as subfamily0 to subfamily13 [19]. Each of these subfamilies has an independent consensus sequence based on sequence alignments, but would be difficult to distinguish otherwise.

### 1.3. Alu Evolution in Platyrrhini

As noted above, the first platyrrhine-specific *Alu* subfamilies identified were Ta7, Ta10 and Ta15 [15]. The first completed WGS for a platyrrhine primate was for the common marmoset (*C. jacchus*; [caljac3]) [22]. Full length *Alu* elements from the marmoset genome were compared to human [hg19], chimpanzee [panTro3], rhesus macaque [rheMac3] and orangutan [ponAbe2] genomes available at that time. Therefore, *Alu* subfamilies discovered in marmoset were referred to as “New World monkey specific” or considered restricted to platyrrhines as viewed thru the lens of the marmoset genome. They were not considered marmoset-specific or even exclusive to Cebidae at that time. Consensus sequences were reported for 94 *Alu* subfamilies from the marmoset genome, called sf0 to sf93 [22]. Following sequence alignment, some overlap with existing *Alu* subfamilies was identified (i.e., sf0 = *Alu*Sx, sf1 = *Alu*Ta15, sf6 = *Alu*Jr, sf13 = *Alu*Sq2 and sf27 = *Alu*Sx1) resulting in a net of *n* = 89 newly identified unique *Alu* subfamilies (Supplementary File S1). One branch of three younger subfamilies, sf4, sf5 and sf43, harbored *Alu* insertion polymorphisms as determined by locus-specific PCR on a DNA sample panel consisting of representative marmosets from three geographically different breeding colonies [22]. Generalized conclusions from these analyses were that platyrrhine *Alu* subfamilies diverge from human and rhesus macaque around the *Alu*Sc branch and that *Alu*Ta15 and its immediate derivatives represent the source of about half of the platyrrhine-specific *Alu* elements [22].

The second completed WGS for a platyrrhine primate was for the squirrel monkey, (*Saimiri boliviensis*; [SaiBol1.0]). Full length *Alu* elements from the *Saimiri* genome were compared against human [hg38], marmoset [calJac3] and owl monkey, genus *Aotus* [Anan_1.0] genomes [18] using the Blast Like Alignment Tool (BLAT) [23]. A combined 108 *Alu* subfamilies derived from both marmoset and squirrel monkey were used for a RepeatMasker custom library to screen the WGS of marmoset and squirrel monkey to determine the *Saimiri* lineage-specific *Alu* subfamilies. Forty-six new *Saimiri Alu* subfamilies were reported, named “Sub_xx_jb” [18] with _jb being the author’s initials. There were fewer new subfamilies (i.e., *n* = 46 compared to *n* = 89) because it was the second platyrrhine genome analyzed and filtered against the first. FASTA consensus sequences for these 46 *Saimiri Alu* subfamilies are available in Supplementary File S2. Generalized conclusions from this study were that *Saimiri Alu* evolution occurred in three major bursts, one each from *Alu*S, *Alu*Ta10 and *Alu*Ta15, with Ta15 and its derived subfamily sf63 comprising the majority of young elements [18].

Each new analyzed genome adds more *Alu* subfamilies unique to that lineage, increasing the complexity of how to report data from these genomic analyses such that a meaningful cross-reference is uniform across taxa. Thus far, the evolution of *Alu* subfamilies in platyrrhines has a structural framework on which to build upon. Starting with the ancestral *Alu*J, *Alu*S and *Alu*Ta-lineages, followed by the sf-lineage discovered in marmoset and then the Sub_xx_jb *Saimiri* subfamilies, a unique naming convention exists on which to build a comprehensive network. In this study we report the *Alu* subfamily composition for two additional genomes, owl monkey, genus *Aotus* [Anan_2.0], and capuchin monkey, genus *Cebus* [Cebus_imitator-1.0], and name them Subfamily_xx_owl and Subfamily_xx_ceb, respectively. Then we combine these new subfamily designations with all of the previous *Alu* subfamilies from RepBase [24], marmoset and squirrel monkey. The goal is to assemble a cohesive framework of the existing *Alu* subfamily network within the three-family clade of Cebidae, Callithrichidae and Aotidae such that the evolving architecture of *Alu* evolution within platyrrhines can be readily integrated in a systematic non-overlapping fashion.

## 2. Materials and Methods

### 2.1. Lineage-Specific Alu Elements

Four high-quality platyrrhine genomes (common marmoset; *C. jacchus* [caljac3], capuchin monkey; *Cebus imitator* [Cebus_imitator-1.0], squirrel monkey; *S. boliviensis* [SaiBol1] and owl monkey; *Aotus nancymaae* [Anan_2.0]) were obtained from the National Center for Biotechnology Information (NCBI) and analyzed for their *Alu* content using RepeatMasker (RepeatMasker-Open-4.0). Ascertainment of lineage-specific or recently integrated *Alu* insertions from the owl monkey genome [Anan_2.0] and from the *C. imitator* genome [Cebus_imitator-1.0] [25] were performed as described previously [26,27,28,29]. Briefly, full-length *Alu* elements were extracted from the RepeatMasker output using a custom python script (described at link https://github.com/t-beck; accessed on 19 December 2022). These elements, along with 600 bp 5′ and 3′ flanking sequence, were then compared to the remaining genomes by means of a sequential BLAT [23] conducted in the following order: (1) human (*Homo sapiens*; [GRCh38.p13]); (2) common marmoset (*C. jacchus*; [caljac3]); (3) capuchin monkey (*C. imitator*; [Cebus_imitator-1.0] or owl monkey (*A. nancymaae*; [Anan_2.0] and (4) squirrel monkey (*S. boliviensis*; [SaiBol1.0]). A sequential BLAT involved analyzing the output after each BLAT for capuchin or owl monkey-specific *Alu* elements compared to the other four genomes.

### 2.2. Alu Subfamily Analysis

The RepeatMasker utility program COSEG was applied to the lineage-specific owl monkey and capuchin monkey *Alu* insertions to determine the subfamily composition based on co-segregating mutations. *Alu* insertions determined to be lineage-specific were aligned via Crossmatch (www.phrap.org/phredphrapconsed.html, accessed on 1 January 2023 with the default settings, then analyzed via COSEG (www.repeatmasker.org/COSEGDownload.html; accessed on 19 December 2022) to determine the subfamily structure. The dataset was aligned against the *Alu*S consensus sequence [30]. COSEG was then used to group the *Alu* subfamilies. The middle A-rich region of the *Alu*S consensus sequence was excluded from the analysis when determining the subfamilies, whereas tri and di segregating mutations were considered. A group of ten or more identical sequences was considered a separate *Alu* subfamily. The consensus sequences were subjected to a RepeatMasker analysis using 24 subfamilies previously defined by RepBase [15,24], as well as the 86 from marmoset and 46 from squirrel monkey (see Section 1) to remove exact matches. Eliminating subfamilies duplicated in owl monkey or capuchin resulted in non-overlapping datasets. These 189 *Alu* subfamilies were then aligned in BioEdit [31] and a network analysis was completed based on the accumulation of diagnostic mutations.

### 2.3. Model Selection

A combined dataset of 189 non-overlapping *Alu* subfamilies was analyzed with jModelTest-2.1.10 [32] to determine the best nucleotide substitution model for this dataset. The Akaike information criterion (AIC) and Bayesian information criterion (BIC) models were a gamma distribution. Both the AIC and BIC model selection were TrN+G (variable base frequencies, equal transversion rates, variable transition rates, and gamma distributed rate variation among sites). The TrN+G model is in agreement with a previous analysis of *Alu* subfamilies derived from the squirrel monkey genome [18].

### 2.4. SCULU Analysis

Subfamilies were analyzed using a new method designed to increase subfamily annotation reliability, implemented in software called Subfamily clustering using label uncertainty (SCULU) [33]. The guiding principle of SCULU is that subfamilies should be reliably separable—if a transposable element insertion properly belongs to one subfamily, then it should be very unlikely to be assigned to some other subfamily due to common chance events. As input, SCULU is provided with consensus sequences for a collection of subfamilies belonging to a single primary family, along with a set of instances for each subfamily. SCULU identifies unreliably separable subfamily pairs empirically, by aligning instances of each subfamily to all of the subfamily consensus sequences, and computing a score-based estimate of annotation confidence to each instance–subfamily pair [34]. If a large number of instances indicate low confidence in the separation of two subfamilies, they are merged. The result is a reduced set of subfamilies, with increased expected subfamily annotation reliability.

### 2.5. Bayesian Phylogenetic Analysis

An alignment of the 189 subfamilies was generated using MUSCLE v3.8.31 [35]. BEAST v1.7 (Bayesian Evolutionary Analysis Sampling Trees) [36] was used for Bayesian analysis and informed by using the jModelTest-2.1.10 analysis. All default settings were used, with the following exceptions: site heterogeneity = γ, species tree prior = birth death process, nucleotide model = TrN, and chain length = 30 million.

## 3. Results

### 3.1. Owl Monkey and Capuchin Alu Element Subfamilies

We found approximately 12,089 owl monkey lineage-specific *Alu* insertions in the [Anan_2.0] genome, from a total of 658,009 full-length insertions [27,28], and approximately 9602 capuchin lineage-specific *Alu* insertions in the [Cebus_imitator-1.0] genome, from a total of 617,132 full-length insertions [29]. An initial COSEG analysis of the lineage-specific *Alu* elements in the capuchin genome indicated that there were 16 subfamilies present. However, upon closer analysis of the data, it was observed that some of the subfamilies had very long 3′ A-tails that comprised half of the consensus sequences predicted by COSEG, or a long middle A-rich region. Upon inspection of the COSEG input for capuchin monkey *Alu* insertions, it was found that there were several hundred *Alu* sequences that contained a string of N’s. These sequences were removed and the COSEG analysis was repeated. Thirty and nine subfamilies were obtained from the owl monkey and capuchin monkey COSEG analyses, respectively. However, five owl monkey subfamilies (of thirty) and one capuchin monkey subfamily (of nine) were removed as these were exact matches to other previously defined subfamilies (described in Supplementary File S3; Table S1). Consensus sequences are available in FASTA format for 25 owl monkey and 8 capuchin *Alu* subfamilies in Supplementary Files S4 and S5, respectively.

### 3.2. Alu Subfamily Network Analysis

**Figure 1:**
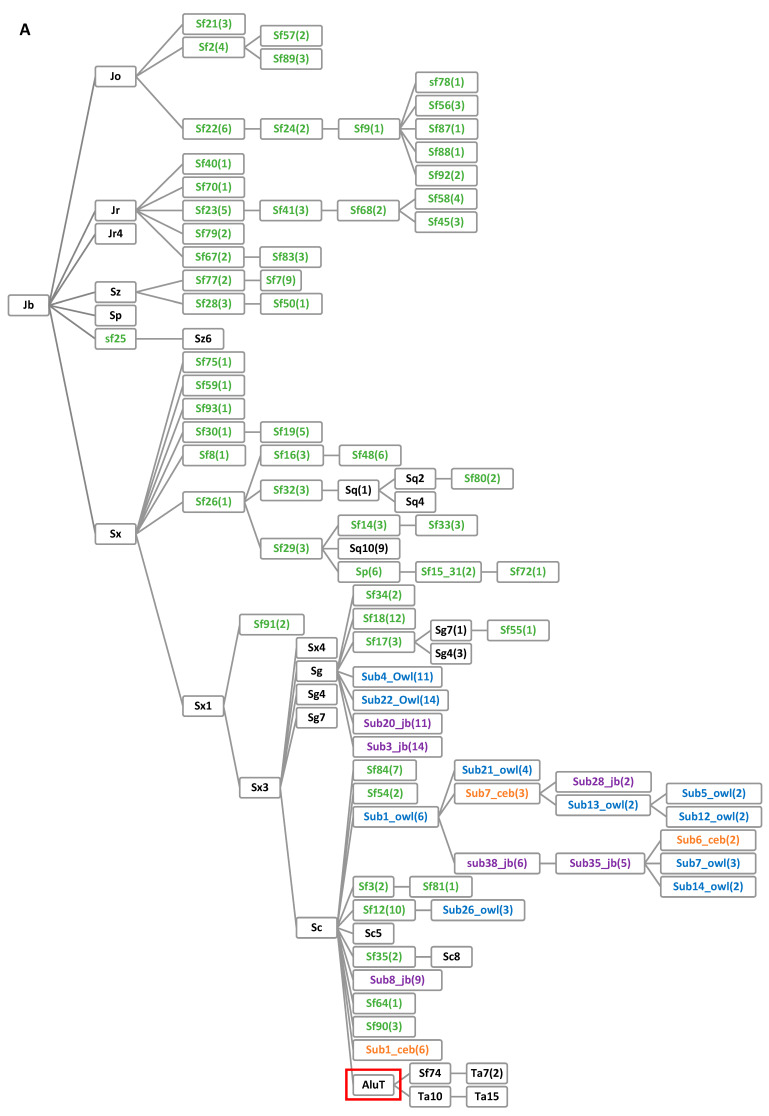

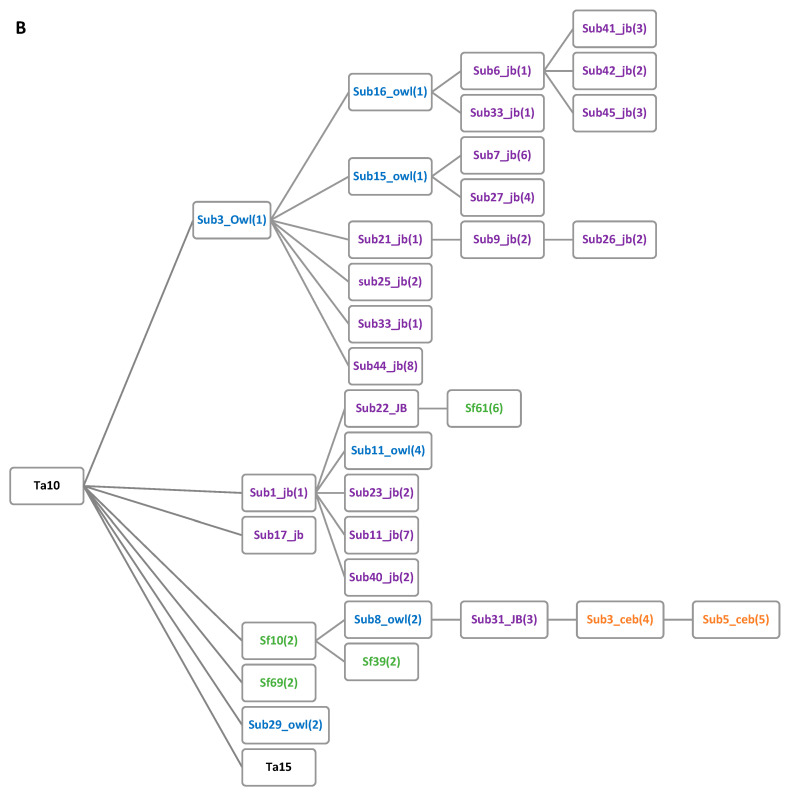

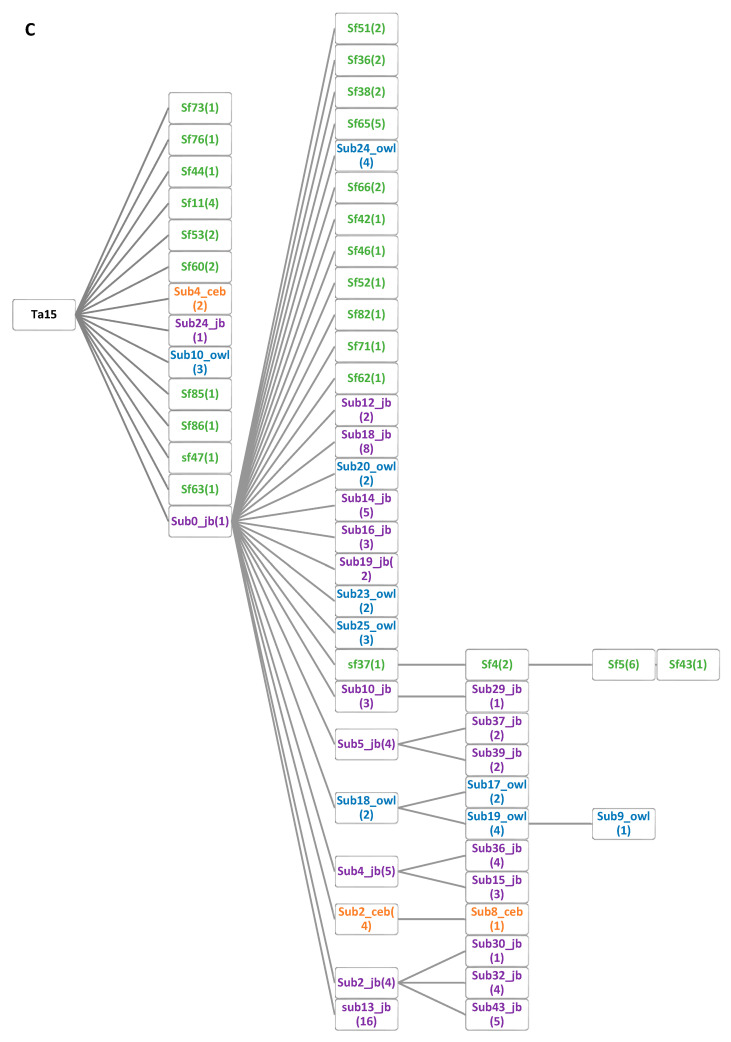
Network schematic of the *Alu* subfamily evolution in the platyrrhine three-family clade of Cebidae, Callithrichidae, and Aotidae. This network was generated using data obtained from RepeatMasker and by a multiple sequence alignment of 189 subfamilies. Black lettering indicates a predefined subfamily [15,24]. Green, purple, blue, or orange lettering indicates that the subfamily was discovered in the marmoset, squirrel monkey, owl monkey, or capuchin monkey genome, respectively. Numbers in parentheses indicate the number of mutations that occurred between the subfamily and its parent node. (**A**) *Alu*J, *Alu*S, and derived subfamilies up to the emergence of *Alu*T. Boxed in red is a putative *Alu*T subfamily as the fusion event between an *Alu*Sc and an *Alu*Sp element [15]. (**B**) *Alu* subfamilies derived from *Alu*Ta10. (**C**) Subfamilies derived from *Alu*Ta15.

A total of 189 unique *Alu* subfamilies were used to generate a network analysis based on the stepwise accumulation of diagnostic mutations (Figure 1). These subfamilies included 24 previously defined by RepBase and Ray and Batzer (2005) [15,24], plus those derived from COSEG analyses of the marmoset, squirrel monkey, owl monkey, and capuchin monkey genomes, contributing 86, 46, 25, and 8 subfamilies, respectively. A RepeatMasker analysis of the 189 subfamilies was used to identify which of four major *Alu* subfamilies (J, S, Ta10, and Ta15) each of the 189 subfamilies were derived from (Supplementary File S3; Table S2). The only *Alu* subfamilies that were grouped with any *Alu*J subfamily (Jb, Jo, Jr, and Jr4) or older *Alu*S subfamilies (Sp, Sq, Sq2, Sq10, Sx, Sx1, Sx3, Sx4, Sz, and Sz6) were ascertained from the marmoset genome (‘sf’) (Figure 1A), the first of the platyrrhine genomes analyzed. Branches with intermediate *Alu*S subfamilies (Sg, Sg4, and Sg7) contain subfamilies from the marmoset as well as squirrel monkey and owl monkey (Figure 1A). The youngest *Alu*S subfamily branch, Sc, contains subfamily representatives from all four of the genomes sequentially analyzed (marmoset, squirrel monkey, owl monkey, and capuchin monkey) and culminates with the emergence of the *Alu*Ta lineage (Figure 1A). The *Alu*Ta10 group was highly represented by lineage-specific squirrel monkey subfamilies, but also included subfamilies ascertained from all four genomes (Figure 1B). The *Alu*Ta15 network has high contributions from both the marmoset and squirrel monkey subfamilies, with lower numbers of lineage-specific owl monkey and capuchin monkey subfamilies (Figure 1C). The branching patterns in the network diagram include several polytomies in which multiple subfamilies are equally related to their parental node, often due to a single uniquely variable nucleotide substitution (Figure 1).

### 3.3. Alu Subfamily Phylogenetic Analysis

**Figure 2:**
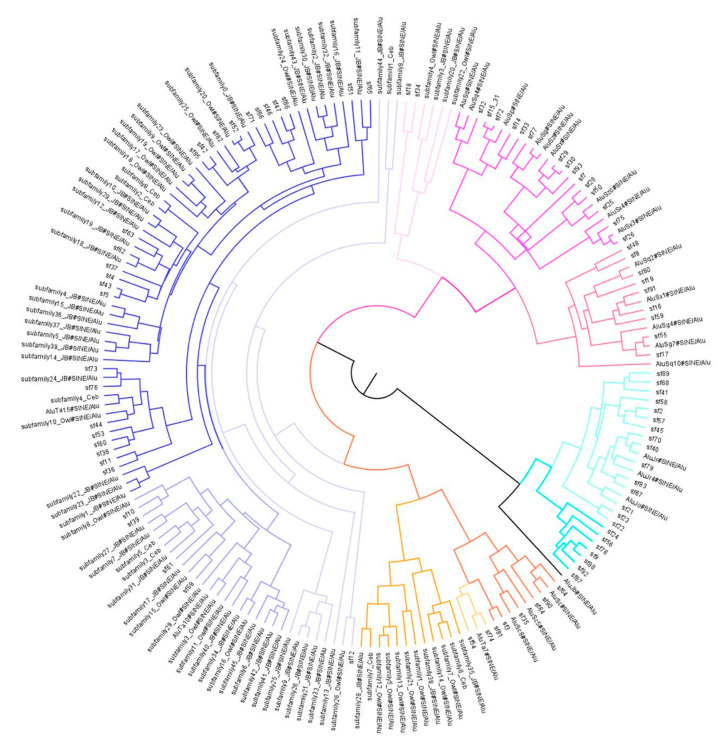
Bayesian tree of *Alu* subfamily evolution for 189 subfamilies. Labels that begin with “sf” are subfamilies identified in the marmoset genome [22]. Labels that contain “jb”, “owl”, and “ceb” were obtained from the squirrel monkey [18], owl monkey, and capuchin monkey, respectively. Teal indicates the *Alu*J branch, with darker shades indicating a basal branch and closer to *Alu*Jb (shown in black), and lighter shades containing Jo and Jr subfamilies. Orange indicates the *Alu*Sc branch, with darker shades corresponding to those subfamilies identified as *Alu*Sc, with lighter shades for those subfamilies with higher percent divergence values forming a separate monophyletic group. Pink in the upper right depicts the older *Alu*S subfamilies, with darker and lighter shades based on sequence divergence values. Purple indicates the *Alu*Ta10 and *Alu*Ta15 subfamilies, with the darkest shade belonging to the branch that contains *Alu*Ta15 (upper left), the lighter shades belonging to *Alu*Ta10 (lower left) and the lightest shades belonging to those subfamilies that form clades but do not form a group within the *Alu*Ta10 or *Alu*Ta15 consensus sequences.

A complementary Bayesian analysis of the evolution of these 189 subfamilies is shown in Figure 2. Polytomies observed in the network diagram are fully resolved in the Bayesian tree due to forced bifurcation [37]. Otherwise, the Bayesian analysis is in general agreement with the RepeatMasker analysis (Supplementary File S3; Table S2) and the network analysis (Figure 1) for the major subfamily groupings. In the *Alu*J portion of the Bayesian tree, it appears as though *Alu* subfamilies Jo and Jr are more closely related than previously thought, as subfamilies identified as Jo or Jr in the RepeatMasker analysis are grouped together in monophyletic branches. However, it should be noted that Jo was generally the basal group in a branching pattern and the only *Alu* subfamilies located within the *Alu*J portion of the Bayesian tree were ascertained from the marmoset genome and labeled “sf”. The *Alu*S portion of the Bayesian tree is also in agreement with the network analysis in terms of certain subfamilies forming a monophyletic group. In addition, there was a separation of *Alu*Sc from all other *Alu*S subfamilies. It was observed that subfamilies with a low percent divergence from the RepeatMasker identified consensus sequence were more likely to have congruent branching patterns with the network analysis, while higher divergence values from the RepeatMasker identified subfamily resulted in less agreement with the network analysis (Supplementary File S3; Table S2, Figure 1). In addition, there is a close relationship between sf74 and *Alu*Ta7, which were placed in the *Alu*Sc group consistently. Subfamilies identified as *Alu*Sz formed a monophyletic group with the RepBase consensus sequence of *Alu*Sz. This same observation was made for the *Alu*Sg7 and *Alu*Sp identified subfamilies. However, the Bayesian analysis and the network analysis did not match when comparing the Sx, Sx1, Sz, and Sq subfamilies in terms of branching pattern and grouping (Figure 1 and Figure 2). This indicates that these subfamilies are potentially more closely related, making exact subfamily designation and branching difficult to determine.

### 3.4. Computationally Distinct Subfamilies

As some of the subfamilies described differ by only a few mutations, with several bearing single nucleotide differences between consensus sequences, we analyzed subfamilies to identify cases in which ambiguity was likely in the assignment of a genomic insertion to a subfamily. This analysis was performed with in-house software called SCULU, which merges subfamilies showing low reliable separability (see Section 2).

This analysis produced a total of 98 subfamilies, with 49 of the original 189 remaining unmerged. Of the 42 merged groups, 25 merged only two subfamilies (Supplementary File S6). The two merged groups with the largest number of members (merged_97 and merged_98 with 10 and 19 members, respectively) included those most closely related to *Alu*Ta15 (Supplementary File S7). This is consistent with the large number of subfamilies with only one or two diagnostic mutations separating them, as well as the large burst of apparent *Alu*Ta15 activity within platyrrhines (Figure 1; Supplementary File S7). For at least five of the merged groups there was a mixture of older *Alu* subfamilies, likely because the copies used as the input for SCULU were more degraded, leading to merged groups. Alternatively, due to the burst of activity in *Alu*Ta10/Ta15 in the platyrrhines, there was minimal difference between active groups, and COSEG potentially made a disproportionate number of *Alu*Ta15-type subfamilies, which were merged by SCULU as well (Supplementary File S7). Therefore, when using this dataset as a library for genomic annotation, great care must be taken to correctly assign genomic insertions to the appropriate subfamily, particularly if they belong to one of the aforementioned merged groups.

### 3.5. Number of Lineage Specific Alu Elements by Subfamily

Characterization of *Alu* subfamilies from marmoset, squirrel monkey, owl monkey, and capuchin were performed in the order of genome availability and thus the “sf” subfamilies obtained from the marmoset appeared to cover a broader range of older and younger subfamilies within the assembled framework of original 189 subfamilies. To determine if this translated to marmoset being the most basal lineage, or whether simply because marmoset was the first of the four to be analyzed for *Alu* content, we analyzed each of the four genomes for full-length *Alu* elements and compared them to the other three genomes to determine lineage specificity (see Methods Section 2.1 regarding Sequential BLAT). Next, we performed an in-house RepeatMasker analysis using our newly constructed repeat library of 189 subfamilies (Supplementary File S8). These results are shown in Supplementary File S3, Table S3, and are summarized in Supplementary File S3, Table S4. The most active subfamilies in these lineages with the highest copy number, such as sf44, sf46, sf47, sf63, sf73, sf82, and sf86, were all Ta15 derived (Supplementary File S3; Table S3, in bold font) and constituted the bulk of active drivers across these four genomes. *Alu*Ta15 and derived subfamilies constituted the vast majority of lineage-specific *Alu* elements in all four of these genomes (Supplementary File S3, Table S4 and Figure S1A). They dominated all other subfamily groups by many thousand-fold. Therefore, to reduce the degree to which *Alu*Ta15 overshadowed other subfamilies, we constructed a separate graph in which data for Ta10 and Ta15 were omitted (Supplementary File S3; Figure S1B). This provides a better view of the limited number of lineage specific insertions from older *Alu*S and *Alu*Ta7 subfamilies. Owl monkey had more lineage-specific *Alu* elements from the oldest *Alu*S subfamilies, 81 compared with 17 in marmoset, and from the young *Alu*S subfamilies, 390 compared to 133 in marmoset (Supplementary File S3, Table S4 and Figure S1B). The number of lineage-specific insertions from *Alu*Ta7 and *Alu*Ta10 were the highest in marmoset and gradually declined in owl monkey, squirrel monkey, and capuchin (Supplementary File S3; Figure S1). These data suggest that owl monkey is likely basal to marmoset.

## 4. Discussion and Conclusions

This *Alu* subfamily analysis provides insight into the evolution of the platyrrhine parvorder by tracking which subfamilies were active in which genomes over time. This study is the first attempt to complete a network of *Alu* subfamilies compiled from multiple datasets of closely-related taxa, and to provide a framework on which to build upon that accommodates large expansions of *Alu* element subfamilies. It is imperative that we pre-emptively prohibit the nomenclature from cascading out of recognizable control in future publications as more WGS are analyzed. Each new analyzed genome adds more *Alu* subfamilies unique to that lineage, increasing the complexity of how to report data from these genomic analyses, such that a meaningful cross-reference with corresponding consensus sequence is uniform for future research. New genome assemblies are rapidly coming online, both within callithrichids, such as *Saguinus imperator* (tamarin), GCA_004024885.1, [SagImp_v1_BIUU] and *Saguinus midas* (Midas tamarin), GCA_021498475.1, [ASM2149847v1], as well as from other platyrrhine branches in Atelidae (*Ateles geoffroyi*, black-handed spider monkey, GCA_004024785.1, [AteGeo_v1_BIUU] and *Alouatta palliata*, mantled howler monkey, GCA_004027835.1, [AloPal_v1_BIUU] and the Pitheciidae branch, such as (*Pithecia pithecia*, white-faced saki, GCA_004026645.1, [PitPit_v1_BIUU] and *Plecturocebus donacophilus*, Bolivian titi, GCA_004027715.1, [CalDon_v1_BIUU].

It seems imperative at this stage to assemble a cohesive framework of the existing *Alu* subfamily structure such that the evolving architecture can be compiled together in a systematic fashion. Otherwise, the starburst-like pattern of the *Alu* subfamily mobilization and rapid rate of expansion in platyrrhines will soon lead to immense confusion. A standardized nomenclature exists; however, it has grown nearly obsolete with the ability to analyze entire genomes in just a few hours, resulting in the simultaneous discovery of multiple overlapping subfamilies. Thus, there are currently too many existing subfamilies to use the standardized nomenclature, mainly because (1) *Alu* subfamily names would become exceedingly long with complicated formatting, and (2) many *Alu* subfamilies have already been published with their corresponding consensus sequences [18,22]. Therefore, it seems prudent to use the consensus sequence as the new standardized metric to avoid naming duplications across multiple taxa. The first platyrrhine genome assembly to be analyzed had the newest *Alu* subfamilies, with decreasing unique consensus sequences in the emerging species. We propose a naming convention be adopted in order to (1) clearly indicate the genome in which the *Alu* subfamily was discovered (i.e., _Ceb for Cebus); (2) include the number of mutations different from the parent subfamily node (as shown in parenthesis in the network diagram, Figure 1); (3) provide the consensus sequence within the context of the congregated framework of 189 *Alu* subfamilies outlined in this report.

These data show bursts of activity arising from the *Alu*Ta10 and *Alu*Ta15 subfamilies, in agreement with the bush-like model of *Alu* mobilization [38,39,40]. The minimal differences between the *Alu*Ta15 consensus sequence and the large number of lineage-specific subfamilies from the marmoset, squirrel monkey, and owl monkey genomes are seen in the few, at times only one, diagnostic mutations. Therefore, the computational method SCULU based on subfamily clustering algorithms was applied to better refine some of these nearly-identical consensus sequences into merged clusters. This study also highlights the concurrent activity of multiple *Alu* subfamilies within each given lineage, similar to what has been previously reported for human-specific *Alu* subfamilies [38]. This is also potentially why the network analysis and phylogenetic tree contained some differences. The diagnostic mutation accumulation may lead to a polytomy, which are avoided in a Bayesian analysis that forces bifurcation [37]. It is also interesting to note that in the *Alu*Ta15 group there is an absence of a hierarchical scheme of lineage-specific subfamilies leading from one organism giving rise to another lineage-specific subfamily to another, as seen in *Alu*Ta10. This provides support that *Alu*Ta15 derived subfamilies are the youngest group of expanding subfamilies within the platyrrhine three-family clade of Cebidae, Callithrichidae, and Aotidae, as previously reported [15].

The analysis of *Alu* elements ascertained from the marmoset, squirrel monkey, capuchin monkey, and owl monkey genomes provide strong evidence of incomplete lineage sorting (ILS). ILS is a product of the rapid speciation that occurred within platyrrhines, during which time a large number of *Alu* insertions remained polymorphic within the emerging taxa and became randomly distributed among the four lineages studied here. Extensive ILS results in incongruent phylogenetic trees [15,37,41,42].

## Data Availability

The algorithms used in this study are available on GitHub (https://github.com/t-beck; accessed on 19 December 2022). The Supplementary Data files are available on the online version of this paper and through the Batzer Lab website under publications, https://biosci-batzerlab.biology.lsu.edu/; accessed on 19 December 2022.

## Supplementary Materials

The following supporting information can be downloaded at: https://www.mdpi.com/2073-4425/14/2/249/s1. Supplementary File S1 contains the *Alu* consensus sequences previously identified in marmoset as well as canonical *Alu* subfamilies (e.g., *Alu*Sc). Supplementary File S2 contains the squirrel monkey *Alu* consensus sequences in fasta format that were previously identified (prior to redundancy reduction). Supplementary File S3 is an excel file containing Tables S1–S4 and Figure S1. Supplementary Files S4 and S5 contains the owl and cebus monkey consensus sequences, respectively, prior to redundancy removal. Supplementary File S6 contains the output of the SCULU program in Newick format (showing which subfamilies have been merged, and in what order), while Supplementary File S7 is the subfamily breakdown of the groups contained within the SCULU output. Supplementary File S8 is the set of 189 *Alu* subfamilies, after redundancy removal, in fasta format. This includes the newly-discovered subfamilies as well as previously-defined subfamilies.
