# Supplementary material for: Framework of the Alu Subfamily Evolution in the Platyrrhine Three-Family Clade of Cebidae, Callithrichidae, and Aotidae
Source: Genes (Basel). 2023 Jan 18;14(2):249. doi: 10.3390/genes14020249 (PMC9956951; doi:10.3390/genes14020249)
Supplement: Supplementary file 1 [file genes-14-00249-s001.zip › Supplementary File S1.pdf]

>AluSx_sf0 count=9363 pvalue=5e-2242 (parent_pvalue=5e-2242) Linup-refined
GGCCGGGCGCGGTGGCTCACGCCTGTAATCCCAGCACTTTGGGAGGCCGAGGCGGGCGGATCACCTGAGGTCA
GGAGTTCGAGACCAGCCTGGCCAACATGGTGAAACCCCGTCTCTACTAAAAATACAAAAATTAGCCGGGCGTG
GTGGCGCGCGCCTGTAATCCCAGCTACTCGGGAGGCTGAGGCAGGAGAATCGCTTGAACCCGGGAGGCGGAGG
TTGCAGTGAGCCGAGATCGCGCCACTGCACTCCAGCCTGGGCGACAGAGCGAGACTCCGTCTCAAAAAA
>AluTa15_sf1 + 49 count=10760 pvalue=6e-4269 (parent_pvalue=5e-2242) parent=0 muts= 85:g/t 75:t/a Linup-refined
GGCCGGGCGCGGTGGCTCACGCCTGTAATCCCAGCACTTTGGGAGGCCGAGGCGGGTGGATCACGAGGTCAAG
AGATCGAGACCATCCTGGTCAACATGGTGAAACCCCGTCTCTACTAAAAATACAAAAAATTAGCTGGGCATGG
TGGCGCGTGCCTGTAATCCCAGCTACTCAGGAGGCTGAGGCAGGAGAATTGCCTGAACCCAGGAGGCGGAGGT
TGCGGTGAGCCGAGATCGCGCCATTGCACTCCAGCCTGGGTAACAAGAGCGAAACTCCGTCTCAAAAAA
>sf2 count=631 pvalue=6e-158 (parent_pvalue=5e-2242) parent=0 muts= 192:a/g 91:c/g Linup-refined
GGCCGGGCGCGGTGGCTCACGCCTGTAATCCCAGCACTTTGGGAGGCCGAGGCGGGAGGATCGCTTGAGCCCA
GGAGTTCGAGACCAGCCTGGGCAACATAGCGAGACCCCGTCTCTACAAAAAATACAAAAAATTAGCCGGGCGT
GGTGGCGCGCGCCTGTAGTCCCAGCTACTCGGGAGGCTGAGGCGGGAGGATCGCTTGAGCCCGGGAGGTCGAG
GCTGCAGTGAGCTGTGATCGCGCCACTGCACTCCAGCCTGGGCGACAGAGCGAGACCCTGTCTCAAAAAA
>sf3 count=5992 pvalue=4e-6183 (parent_pvalue=6e-4269) parent=1 muts= 265:g/c 253:c/t Linup-refined
GGCCGGGCGCGGTGGCTCACGCCTGTAATCCCAGCACTTTGGGAGGCCGAGGCGGGCGGATCACGAGGTCAAG
AGATCGAGACCATCCTGGCCAACATGGTGAAACCCCGTCTCTACTAAAAATACAAAAATTAGCTGGGCGTGGT
GGCGCGCGCCTGTAGTCCCAGCTACTCGGGAGGCTGAGGCAGGAGAATCGCTTGAACCCGGGAGGCGGAGGTT
GCAGTGAGCCGAGATCGCGCCACTGCACTCCAGCCTGGGCGACAGAGCGAGACTCCGTCTCAAAAAA
>sf4 count=588 pvalue=1e-260 (parent_pvalue=6e-4269) parent=1 muts= 209:a/g 72:g/c Linup-refined
GGCCGGGCGCGGTGGCTCAAGCCTGTAATCCCAGCACTTTGGGAGGCCGAGGCGGGTGGATCACGAGGTCAAC
AGATCGAGACCATCCTGGTCAACATGGTGAAACCCCGTCTCTACTAAAAATTACAAAAAATTAGCTGGGCACG
GTGGCGCGTGCCTGTAATCCCAGCTACTCAGGAGGCTGAGGCAGGAGAATTGCCTGAACCCAGGGGGCGGAGG
TTGCGGTGAGCCGAGATCGCGCCATTGCACTCCAGCCTGGGTAACAAGAGCGAAACTCCGTCTCAAAAAA
>sf5 count=189 pvalue=1e-119 (parent_pvalue=6e-4269) parent=1 muts= 170:a/g 87:c/t Linup-refined
GGCCGGGCGCGGTGGCTCAAGCCTGTAATCCCAGCACTTTGGGAGGCCGAGGCGGGTGGATCACGAGGTCAAC
AGATCGAGACCATCTTGGTCAACATGGTGAAACCCCGTCTCTACTAAAATTACAAAAAATTAGCTGGGCACGG
TGGTGCGTGCCTGTAATCCCAGCTGCTCGGGAGGCTGAGGCAGGAGAATTGCCTGAACCCAGGAGGCGGAGAT
TGCGGTGAGCCGAGATCGCGCCATTGCACTCCAGCCTGGGTAACAAGAGCGAAACTCCGTCTCAAAAAA
>AluJr_sf6 + 20 count=1642 pvalue=6e-158 (parent_pvalue=6e-158) parent=2 muts= 282:a/t 211:g/t Linup-refined
GGCCGGGCGCGGTGGCTCACGCCTGTAATCCCAGCACTTTGGGAGGCCGAGGCGGGAGGATCGCTTGAGGCCA
GGAGTTCGAGACCAGCCTGGGCAACATAGCGAGACCCCGTCTCTACAAAAAATATAAAAAATTAGCCGGGCGT
GGTGGCGCGCGCCTGTAGTCCCAGCTACTCGGGAGGCTGAGGCGGGAGGATCGCTTGAGCCCAGGAGTTCGAG
GCTGCAGTGAGCTATGATCGCGCCACTGCACTCCAGCCTGGGCGACAGAGCGAGACCCTGTCTCAAAAAA
>sf7 count=146 pvalue=4e-21 (parent_pvalue=5e-2242) parent=0 muts= 188:g/c 101:g/a Linup-refined
AGCCGGGCGCGGTGGCTCACGCCTGTAATCCCAGCACTTTGGGAGGCCGAGGCGGGCAGATCACTTGAGGTCA
GGAGTTCGAGACCAGCCTGGCCAACATGGTAAAACCCCGTCTCTACTAAAAATACAAAAATTAGCCAGGTGTG
GTGGTGCACGCCTGTAATCCCAGCTACTCGGGAGGCTGAGGCACAAGAATCGCTTGAACCCGGGAGGCGGAGG
TTGCAGTGAGCCGAGATCGCGCCACTGCACTCCAGCCTGGGCGACAGAGCGAGACTCCGTCTCAAAAAA
>sf8 count=493 pvalue=2e-48 (parent_pvalue=5e-2242) parent=0 muts= 145:g/a 117:t/- Linup-refined
GGCCGGGCGCGGTGGCTCACGCCTGTAATCCTAGCACTTTGGGAGGCCGAGGCGGGCGGATCACCTGAGGTCA
GGAGTTCGAGACCAGCCTGGCCAACATGGTGAAACCCCGTCTCTACTAAAAATACAAAAATTAGCCGGGCGTG
GTGGCGCGCGCCTGTAATCCCAGCTACTCGGGAGGCTGAGGCAGGAGAATCGCTTGAACCCGGGAGGCGGAGG
TTGCAGTGAGCCGAGATCGCGCCACTGCACTCCAGCCTGGGCGAAAGAGCGAAACTCCGTCTCAAAAAA

>sf9 count=1242 pvalue=1e-5 (parent_pvalue=5e-2242) parent=0 muts=
61:c/t 31:c/t Linup-refined
GGCCGGGCGCGGTGGCTCACGCCTGTAATCCCAGCACTTTGGGAGGCCGAGGCGGGCGGATCGCTTGAGCCCA
GGAGTTCGAGACCAGCCTGGGCAACATGGCGAAACCCCGTCTCTACAAAAAATACAAAAAATTAGCCGGGCGT
GGTGGCGCGCGCCTGTAGTCCCAGCTACTCGGGAGGCTGAGGTGGGAGGATCGCTTGAGCCCGGGAGGTCGAG
GCTGCAGTGAGCCGTGATCGCGCCACTGCACTCCAGCCTGGGCGACAGAGCGAGACCCTGTCTCAAAAAA
>sf10 count=2609 pvalue=3e-1473 (parent_pvalue=6e-4269) parent=1 muts=
265:g/c 253:c/t Linup-refined
GGCCGGGCGCGGTGGCTCACGCCTATAATCCCAGCACTTTGGGAGGCCGAGGCGGGTGGATCACGAGGTCAAG
AGATCGAGACCATCCTGGTCAACAAGGTGAAACCCCGTCTCTACTAAAAATACAAAAATTAGCTGGGCATGGT
GGCGCGCGCCTGTAGTCCCAGCTACTCGGGAGGCTGAGGCAGGAGAATTGCTTGAACCCAGGAGGCGGAGGTT
GCGGTGAGCCGAGATCGCGCCATTGCACTCCAGCCTGGGTAACAACAGCGAAACTCCGTCTCAAAAAA
>sf11 count=132 pvalue=2e-66 (parent_pvalue=6e-4269) parent=1 muts=
88:t/a 51:g/a Linup-refined
GGCCGGGCGCGGTGGCTCACGCCTGTAATCCCAGCACTTTGGGAGGCCGAGACGGGTGGATCACGAGGTCAAG
AGATCGAGACCATCCAGGTCAACATGGTGAAACCCCGTCTCTACTAAAAATACAAAAAATTAGCTGGGCATGG
TGGCGCGTGCCTGTAATCCCAGCTACTCGGGAGGCTGAGGCAGGAGAATTGCCTGAGCCCAGGAGGCGGAGGT
TGCGGTGAGCCGAGATCGCGCCATTGCACTCCAGCCTGGGTAACAAGAGCGAAACTCCGTCTCAAAAAA
>sf12 count=164 pvalue=3e-83 (parent_pvalue=4e-6183) parent=3 muts=
216:g/a 203:c/a Linup-refined
GGCCGGGCGCGGTGGCTCACGCCTGTAATCCCAGCACTTTGGGAGGCCGAGGCGGGCGGATCACGAGGTCAAG
AGATCGAGACCATCCTGGCCAACATGGTGAAACCCCGTCTCTACTAAAAATACAAAAAATTAGCCGGGCGTGG
TGGTGCGTGCCTGTAGTCCCAGCTACTCGGGAGGCTGAGGCAGGAGAATTGCTTGAAACCGGAAAACGGAAGT
TGCAGTGAGCCGGGATCGCGCCACTGCACTCCAGCCTGGCGATAGAGCGAGACTCCGTCTCAAAAAA
>AluSq2_sf13 count=2987 pvalue=9e-1230 (parent_pvalue=6e-4269) parent=1
muts= 234:t/a 185:g/a Linup-refined
GGCCGGGCGCGGTGGCTCACGCCTGTAATCCCAGCACTTTGGGAGGCCGAGGCGGGCGGATCACCTGAGGTCA
GGAGTTCGAGACCAGCCTGGCCAACATGGTGAAACCCCGTCTCTACTAAAAATACAAAAATTAGCCGGGCGTG
GTGGCGGGCGCCTGTAATCCCAGCTACTCGGGAGGCTGAGGCAGGAGAATCGCTTGAACCCGGGAGGCGGAGG
TTGCAGTGAGCCGAGATCGCGCCATTGCACTCCAGCCTGGGCGACAAGAGCGAAACTCCGTCTCAAAAAA
>sf14 count=453 pvalue=4e-48 (parent_pvalue=9e-1230) parent=13 muts=
111:c/t 92:c/t Linup-refined
GGCCGGGCGCGGTGGCTCACGCCTGTAATCCCAGCACTTTGGGAGGCCGAGGCGGGCGGATCACCTGAGGTCA
GGAGTTCGAGACCAGCCTGGCCAACATGGTGAAACCCCGTTTCTACTAAAAATACAAAAAATTAGCCGGGCGT
GGTGGCGCGCGCCTGTAATCCCAGCTACTCGGGAGGCTGAGGCAGGAGAATCGCTTGAACCCGGGAGGCGGAG
GTTGCAGTGAGCCGAGATCGCGCCATTGCACTCCAGCCTGGGCAACAAGAGCGAAACTCCGTCTCAAAAAA
>sf15 + 31 count=4817 pvalue=6e-1473 (parent_pvalue=4e-48) parent=14
muts= 100:t/a 90:g/a Linup-refined
GGCCGGGCGCGGTGGCTCACGCCTGTAATCCCAGCACTTTGGGAGGCCGAGGCGGGCGGATCACCTGAGGTCG
GGAGTTCGAGACCAGCCTGACCAACATGGAGAAACCCCGTCTCTACTAAAAATACAAAATTAGCCGGGCGTGG
TGGCGCATGCCTGTAATCCCAGCTACTCGGGAGGCTGAGGCAGGAGAATCGCTTGAACCCGGGAGGCGGAGGT
TGCGGTGAGCCGAGATCGCGCCATTGCACTCCAGCCTGGGCAACAAGAGCGAAACTCCGTCTCAAAAAA
>sf16 count=4343 pvalue=8e-89 (parent_pvalue=9e-1230) parent=13 muts=
262:c/- 31:c/t Linup-refined
GGCCGGGCGCGGTGGCTCACGCCTGTAATCCCAGCACTTTGGGAGGCCGAGGCGGGCGGATCACCTGAGGTCA
GGAGTTCGAGACCAGCCTGGCCAACATGGTGAAACCCCGTCTCTACTAAAAATACAAAAATTAGCCGGGCGTG
GTGGCGGGCGCCTGTAATCCCAGCTACTCGGGAGGCTGAGGCAGGAGAATCGCTTGAACCCGGGAGGCGGAGG
TTGCAGTGAGCCGAGATCGCGCCACTGCACTCCAGCCTGGGCGACAAGAGCGAGACTCCGTCTCAAAAAA
>sf17 count=850 pvalue=2e-300 (parent_pvalue=8e-89) parent=16 muts=
189:g/- 95:c/g Linup-refined
GGCCGGGCGCGGTGGCTCACGCCTGTAATCCCAGCACTTTGGGAGGCCGAGGCGGGTGGATCACGAGGTCAGG
AGTTCAAGACCAGCCTGGCCAAGATGGTGAAACCCCGTCTCTACTAAAAATACAAAAATTAGCCGGGCGTGGT
GGCGGGCGCCTGTAATCCCAGCTACTCGGGAGGCTGAGGCAGAGAATCGCTTGAACCCGGGAGGCGGAGGTTG
CAGTGAGCCGAGATCGCGCCACTGCACTCCAGCCTGGGCGACAGAGCGAGACTCCGTCTCAAAAAA

>sf18 count=158 pvalue=6e-101 (parent_pvalue=5e-2242) parent=0 muts=
260:g/t 86:c/a Linup-refined
GGCCGGGCGCGGTGGCTCACGCCTGTAATCCCAGCACTTTGGGAGGCCGAGGCGGGCGGATCACGAGGTCAGG
AGTTCAAGACCAGACTGGCCAACATGGTGAAACCCCGTCTCTACTAAAAAATACAAAAATTAGCTGGGCATGG
TGGCTCGTGCCTGTAATCCCAGCTACTTGGGAGGCTGAGGTAGGAGAATTGCTTGAACCCGGGAGGCGGAGGT
TGCAGTGAGCCGAGATCGCGCCACTGCACTCCAGCCTGGGCTACAGAGCGAGACTCCATCTCAAAAAA
>sf19 count=99 pvalue=7e-50 (parent_pvalue=5e-2242) parent=0 muts=
145:g/a 117:t/- Linup-refined
GGCCGGGCGCGGTGGCTCACGCCTGTAATCCCAGCACTTTGGGAGGCCGAGGCGGGCGGATCACCTGAGGTCA
GGAGTTCAAGACCAGCCTGGCCAACATGGTGAAACCCCGTCTCTACAAAAATACAAAAATTAGCCGGGCATGA
TGGCGGGTGCCTGTAATCCCAGCTACTCGGGAGGCTGAGGCGGGAGAATCGCTTGAACCCGGGAGGCGGAGGT
TGCAGTGAGCCGAGATCGCGCCATTGCACTCCAGCCTGGGCGACAGAGCGAGACTCCGTCTCAAAAAA
>sf21 count=297 pvalue=4e-54 (parent_pvalue=6e-158) parent=6 muts=
67:g/c Linup-refined
GGCTGGGCGCAGTGGCTCACGCCTGTAATCCCAGCACTTTGGGAGGCCGAGGCGGGAGGATCGCTTGAGCCCA
GGAGTTCGAGACCAGCCTGGGCAACATAGCGAGACCCCGTCTCTACAAAAAATATAAAAAATTAGCCGGGCGT
GGTGGCGCGCGCCTGTAGTCCCAGCTACTCGGGAGGCTGAGGCAGGAGGATCGCTTGAGCCCAGGAGTTCGAG
GCTGCAGTGAGCTATGATCGCGCCACTGCACTCCAGCCTGGGCGACAGAGCGAGACCCTGTCTCAAAAAA
>sf22 count=1177 pvalue=8e-1433 (parent_pvalue=1e-5) parent=9 muts=
103:a/g Linup-refined
GGCCGGGCGCGGTGGCTCACGCCTGTAATCCCAGCACTTTGGGAGGCCGAGGCGGGAGGATCGCTTGAGCCCA
GGAGTTCGAGACCAGCCTGGGCAACATAGCGAGACCCCGTCTCTACAAAAAATACAAAAAATTAGCCGGGCGT
GGTGGCGCGCGCCTGTAGTCCCAGCTACTCGGGAGGCTGAGGTGGGAGGATCGCTTGAGCCCGGGAGGTCGAG
GCTGCAGTGAGCCGTGATCGCGCCACTGCACTCCAGCCTGGGCGACAGAGCGAGACCCTGTCTCAAAAAA
>sf23 count=1272 pvalue=3e-19 (parent_pvalue=6e-158) parent=2 muts=
229:t/c Linup-refined
GGCCGGGCGCGGTGGCTCACGCCTGTAATCCCAGCACTTTGGGAGGCCGAGGCGGGAGGATCGCTTGAGCCCA
GGAGTTCGAGACCAGCCTGGGCAACATAGCGAGACCCCGTCTCTACAAAAAATATAAAAAATTAGCCGGGCGT
GGTGGCGCGCGCCTGTAGTCCCAGCTACTCGGGAGGCTGAGGTGGGAGGATCGCTTGAGCCCGGGAGGTCGAG
GCTGCAGTGAGCCATGATCGCGCCACTGCACTCCAGCCTGGGCGACAGAGCGAGACCCTGTCTCAAAAAA
>sf24 count=887 pvalue=7e-745 (parent_pvalue=1e-5) parent=9 muts=
56:t/a Linup-refined
GGCCGGGCGCGGTGGCTCACGCCTGTAATCCCAGCACTTTGGGAGGCCGAGGCGGGAGGATCGCTTGAGCCCA
GGAGTTCGAGACCAGCCTGGGCAACATGGCGAAACCCCGTCTCTACAAAAAATACAAAAAATTAGCCGGGCGT
GGTGGCGCGCGCCTGTAGTCCCAGCTACTCGGGAGGCTGAGGTGGGAGGATCGCTTGAGCCCGGGAGGTCGAG
GCTGCAGTGAGCCGTGATCGCGCCACTGCACTCCAGCCTGGGCGACAGAGCGAGACCCTGTCTCAAAAAA
>sf25 count=4205 pvalue=3e-191 (parent_pvalue=5e-2242) parent=0 muts=
68:t/c Linup-refined
GGCCGGGCGCGGTGGCTCACGCCTGTAATCCCAGCACTTTGGGAGGCCGAGGCGGGCGGATCACTTGAGGCCA
GGAGTTCGAGACCAGCCTGGCCAACATGGCGAAACCCCGTCTCTACTAAAAATACAAAAATTAGCCGGGCGTG
GTGGCGCGCGCCTGTAATCCCAGCTACTCGGGAGGCTGAGGCAGGAGAATCGCTTGAACCCGGGAGGCGGAGG
TTGCAGTGAGCCGAGATCGCGCCACTGCACTCCAGCCTGGGCGACAGAGCGAGACTCTGTCTCAAAAAA
>sf26 count=7844 pvalue=9e-77 (parent_pvalue=5e-2242) parent=0 muts=
161:a/g Linup-refined
GGCCGGGCGCGGTGGCTCACGCCTGTAATCCCAGCACTTTGGGAGGCCGAGGCGGGCGGATCACCTGAGGTCA
GGAGTTCGAGACCAGCCTGGCCAACATGGTGAAACCCCGTCTCTACTAAAAATACAAAAATTAGCCGGGCGTG
GTGGCGCGCGCCTGTAGTCCCAGCTACTCGGGAGGCTGAGGCAGGAGAATCGCTTGAACCCGGGAGGCGGAGG
TTGCAGTGAGCCGAGATCGCGCCACTGCACTCCAGCCTGGGCGACAGAGCGAGACTCCGTCTCAAAAAA
>AluSx1_sf27 count=5288 pvalue=4e-276 (parent_pvalue=5e-2242) parent=0
muts= 151:c/g Linup-refined
GGCCGGGCGCGGTGGCTCACGCCTGTAATCCCAGCACTTTGGGAGGCCGAGGCGGGCGGATCACCTGAGGTCA
GGAGTTCGAGACCAGCCTGGCCAACATGGTGAAACCCCGTCTCTACTAAAAATACAAAAATTAGCCGGGCGTG
GTGGCGGGCGCCTGTAATCCCAGCTACTCGGGAGGCTGAGGCAGGAGAATCGCTTGAACCCGGGAGGCGGAGG
TTGCAGTGAGCCGAGATCGCGCCACTGCACTCCAGCCTGGGCGACAGAGCGAGACTCCGTCTCAAAAAA

>sf28 count=805 pvalue=5e-38 (parent_pvalue=5e-2242) parent=0 muts=
188:g/c Linup-refined
GGCCGGGCGCGGTGGCTCACGCCTGTAATCCCAGCACTTTGGGAGGCCGAGGCGGGCGGATCACTTGAGGTCA
GGAGTTCGAGACCAGCCTGGCCAACATGGCGAAACCCCGTCTCTACTAAAAATACAAAAATTAGCCGGGCGTG
GTGGCGCGCGCCTGTAATCCCAGCTACTCGGGAGGCTGAGGCACGAGAATCGCTTGAACCCGGGAGGCGGAGG
TTGCAGTGAGCCGAGATCGCGCCACTGCACTCCAGCCTGGGCGACAGAGCGAGACTCTGTCTCAAAAAA
>sf29 count=3417 pvalue=4e-128 (parent_pvalue=9e-1230) parent=13 muts=
271:a/g Linup-refined
GGCCGGGCGCGGTGGCTCACGCCTGTAATCCCAGCACTTTGGGAGGCCGAGGCGGGCGGATCACCTGAGGTCA
GGAGTTCGAGACCAGCCTGGCCAACATGGTGAAACCCCGTCTCTACTAAAAATACAAAAATTAGCCGGGCGTG
GTGGCGCGCGCCTGTAATCCCAGCTACTCGGGAGGCTGAGGCAGGAGAATCGCTTGAACCCGGGAGGCGGAGG
TTGCAGTGAGCCGAGATCGCGCCATTGCACTCCAGCCTGGGCGACAAGAGCGAGACTCCGTCTCAAAAAA
>sf30 count=2081 pvalue=3e-16 (parent_pvalue=5e-2242) parent=0 muts=
242:c/t Linup-refined
GGCCGGGCGCGGTGGCTCACGCCTGTAATCCCAGCACTTTGGGAGGCCGAGGCGGGCGGATCACCTGAGGTCA
GGAGTTCGAGACCAGCCTGGCCAACATGGTGAAACCCCGTCTCTACTAAAAATACAAAAATTAGCCGGGCGTG
GTGGCGCGCGCCTGTAATCCCAGCTACTCGGGAGGCTGAGGCAGGAGAATCGCTTGAACCCGGGAGGCGGAGG
TTGCAGTGAGCCGAGATCGCGCCATTGCACTCCAGCCTGGGCGACAGAGCGAGACTCCGTCTCAAAAAA
>sf32 count=2231 pvalue=1e-109 (parent_pvalue=9e-1230) parent=13 muts=
242:t/c Linup-refined
GGCCGGGCGCGGTGGCTCACGCCTGTAATCCCAGCACTTTGGGAGGCCGAGGCGGGCGGATCACCTGAGGTCA
GGAGTTCGAGACCAGCCTGGCCAACATGGTGAAACCCCGTCTCTACTAAAAATACAAAAATTAGCCGGGCGTG
GTGGCGCGCGCCTGTAATCCCAGCTACTCGGGAGGCTGAGGCAGGAGAATCGCTTGAACCCGGGAGGCGGAGG
TTGCAGTGAGCCGAGATCGCGCCACTGCACTCCAGCCTGGGCAACAAGAGCGAAACTCCGTCTCAAAAAA
>sf33 count=2082 pvalue=2e-3050 (parent_pvalue=9e-1230) parent=13 muts=
151:g/c Linup-refined
GGCCGGGCGCGGTGGCTCACGCCTGTAATCCCAGCACTTTGGGAGGCCGAGGCGGGCGGATCACCTGAGGTCA
GGAGTTCGAGACCAGCCTGGCCAACATGGTGAAACCCCGTCTCTACTAAAAATACAAAAATTAGCCGGGCGTG
GTGGCGCATGCCTGTAATCCCAGCTACTCGGGAGGCTGAGGCAGGAGAATCGCTTGAACCCGGGAGGCGGAGG
TTGCAGTGAGCCGAGATCGCGCCATTGCACTCCAGCCTGGGCAACAAGAGCGAAACTCCGTCTCAAAAAA
>sf34 count=60 pvalue=1e-4 (parent_pvalue=6e-101) parent=18 muts=
186:t/c Linup-refined
GGCCGGGCGCGGTGGCTCACGCCTGTAATCCCAGCACTTTGGGAGGCCGAGGCGGGCGGATCACGAGGTCAGG
AGTTCAAGACCAGCCTGGCCAACATGGTGAAACCCCGTCTCTACTAAAAATACAAAAATTAGCTGGGCGTGGT
GGCNCGTGCCTGTAATCCCAGCTACTCGGGAGGCTGAGGCAGGAGAATTGCTTGAACCCGGGAGGCGGAGGTT
GCAGTGAGCCGAGATCGCGCCACTGCACTCCAGCCTGGGCTACAGAGCGAGACTCCATCTCAAAAAA
>sf35 count=5739 pvalue=9e-211 (parent_pvalue=4e-6183) parent=3 muts=
71:a/g Linup-refined
GGCCGGGCGCGGTGGCTCACGCCTGTAATCCCAGCACTTTGGGAGGCCGAGGCGGGCGGATCACGAGGTCAGG
AGATCGAGACCATCCTGGCCAACATGGTGAAACCCCGTCTCTACTAAAAATACAAAAAATTAGCCGGGCGTGG
TGGCGCGCGCCTGTAGTCCCAGCTACTCGGGAGGCTGAGGCAGGAGAATCGCTTGAACCCGGGAGGCGGAGGT
TGCAGTGAGCCGAGATCGCGCCACTGCACTCCAGCCTGGGCGACAGAGCGAGACTCCGTCTCAAAAAA
>sf36 count=169 pvalue=5e-25 (parent_pvalue=6e-4269) parent=1 muts=
88:t/a Linup-refined
GGCCGGGCGCGGTGGCTCAAGCCTGTAATCCCAGCACTTTGGGAGGCCGAGGCGGGTGGATCACGAGGTCAAG
AGATCGAGACCATCCAGGTCAACATGGTGAAACCCCGTCTCTACTAAAAATACAAAAAATTAGCTGGGCATGG
TGGCGCGTGCCTGTAATCCCAGCTACTCGGGAGGCTGAGGCAGGAGAATTGCCTGAACCCAGGAGGCGGAGGT
TGCGGTGAGCCGAGATCGCGCCATTGCACTCCAGCCTGGGTAACAAGAGCGAAACTCCGTCTCAAAAAA
>sf37 count=1176 pvalue=5e-1595 (parent_pvalue=6e-4269) parent=1 muts=
72:g/c Linup-refined
GGCCGGGCGCGGTGGCTCAAGCCTGTAATCCCAGCACTTTGGGAGGCCGAGGCGGGTGGATCACGAGGTCAAC
AGATCGAGACCATCCTGGTCAACATGGTGAAACCCCGTCTCTACTAAAAATACAAAAAATTAGCTGGGCATGG
TGGCGCGTGCCTGTAATCCCAGCTACTCAGGAGGCTGAGGCAGGAGAATTGCCTGAACCCAGGAGGCGGAGGT
TGCGGTGAGCCGAGATCGCGCCATTGCACTCCAGCCTGGGTAACAAGAGCGAAACTCCGTCTCAAAAAA

>sf38 count=468 pvalue=4e-198 (parent_pvalue=6e-4269) parent=1 muts=
97:t/a Linup-refined
GGCCGGGCGCGGTGGCTCANGCCTGTAATCCCAGCACTTTGGGAGGCCGAGGCGGGTGGATCACGAGGTCAAG
AGATCGAGACCATCCTGGTCAACAAGGTGAAACCCCGTCTCTACTAAAAATACAAAAAATTAGCTGGGCATGG
TGGCGCGTGCCTGTAATCCCAGCTACTCGGGAGGCTGAGGCAGGAGAATTGCCTGAACCCAGGAGGCGGAGGT
TGCGGTGAGCCGAGATCGCGCCATTGCACTCCAGCCTGGGTAACAAGAGCGAAACTCCGTCTCAAAAAA
>sf39 count=224 pvalue=7e-63 (parent_pvalue=3e-1473) parent=10 muts=
198:t/c Linup-refined
AGCCGGGCGCGGTGGCTCACGCCTATAATCCCAGCACTTTGGGAGGCCGAGGCGGGTGGATCACGAGGTCAAG
AGATCGAGACCATCCTGGTCAACAAGGTGAAACCCCGTCTCTACTAAAAATACAAAAATTAGCTGGGCATGGT
GGCGCGCGCCTGTAGTCCCAGCTACTCGGGAGGCTGAGGCAGGAGAATTGCCTGAACCCAGGAGGCGGAGGTT
GCGGTGAGCCGAGATCGCGCCATTGCACTCCAGCCTGGGTAACAACAGCGAAACTCCGTCTCAAAAAA
>sf40 count=326 pvalue=3e-396 (parent_pvalue=6e-158) parent=6 muts=
282:a/t Linup-refined
GGCCGGGCGCGGTGGCTCACGCCTGTAATCCCAGCACTTTGGGAGGCCGAGGCGGGAGGATCGCTTGAGGCCA
GGAGTTCGAGACCAGCCTGGGCAACATAGCGAGACCCCGTCTCTACAAAAAATATAAAAAATTAGCCGGGCGT
GGTGGCGCGCGCCTGTAGTCCCAGCTACTCGGGAGGCTGAGGCGGGAGGATCGCTTGAGCCCAGGAGTTCGAG
GCTGCAGTGAGCTATGATCGCGCCACTGCACTCCAGCCTGGGCGACAGAGCGAGACCCTGTCTCTAAAAA
>sf41 count=344 pvalue=2e-248 (parent_pvalue=6e-158) parent=2 muts=
124:a/t Linup-refined
GGCCGGGCGCGGTGGCTCACGCCTGTAATCCCAGCACTTTGGGAGGCCGAGGCGGGAGGATCGCTTGAGCCCA
GGAGTTCGAGACCAGCCTGGGCAACATAGCGAGACCCCGTCTCTACAAAAAATTTAAAAAATTAGCCGGGCGT
GGTGGCGCGCGCCTGTAGTCCCAGCTACTCGGGAGGCTGAGGTGGGAGGATCGCTTGAGCCCGGGAGGTCGAG
GCTGCAGTGAGCTGTGATCGCGCCACTGCACTCCAGCCTGGGCGACAGAGCGAGACCCTGTCTCAAAAAA
>sf42 count=378 pvalue=1e-229 (parent_pvalue=6e-4269) parent=1 muts=
74:g/t Linup-refined
GGCCGGGCGCGGTGGCTCAAGCCTGTAATCCCAGCACTTTGGGAGGCCGAGGCGGGTGGATCACGAGGTCAAG
ATATCGAGACCATCCTGGTCAACATGGTGAAACCCCGTCTCTACTAAAAATACAAAAAATTAGCTGGGCATGG
TGGCGCGTGCCTGTAATCCCAGCTACTCAGGAGGCTGAGGCAGGAGAATTGCCTGAACCCAGGAGGCGGAGGT
TGCGGTGAGCCGAGATCGCGCCATTGCACTCCAGCCTGGGTAACAAGAGCGAAACTCCGTCTCAAAAAA
>sf43 count=41 pvalue=4e-229 (parent_pvalue=1e-119) parent=5 muts=
261:a/g Linup-refined
GGCCGGGCGCGGTGGCTCAAGCCTGTAATCCCAGCACTTTGGGAGGCCGAGGCGGGTGGATCACGAGGTCAAC
AGATCGAGACCATCTTGGTCAACATGGTGAAACCCCGTCTCTACTAAAATTACAAAAAATTAGCTGGGCACGG
TGGTGCGTGCCTGTAATCCCAGCTGCTCGGGAGGCTGAGGCAGGAGAATTGCCTGAACCCAGGAGGCGGAGAT
TGCGGTGAGCCGAGATCGCGCCATTGCACTCCAGCCTGGGTAGCAAGAGCGAAACTCCGTCTCAAAAAA
>sf44 count=204 pvalue=2e-127 (parent_pvalue=6e-4269) parent=1 muts=
0:g/c Linup-refined
CGCCGGGCGCGGTGGCTCACGCCTGTAATCCCAGCACTTTGGGAGGCCGAGGCGGGTGGATCACGAGGTCAAG
AGATCGAGACCATCCTGGTCAACATGGTGAAACCCCGTCTCTACTAAAAATACAAAAAATTAGCTGGGCATGG
TGGCGCGTGCCTGTAATCCCAGCTACTCAGGAGGCTGAGGCAGGAGAATTGCCTGAACCCAGGAGGCGGAGGT
TGCGGTGAGCCGAGATCGCGCCATTGCACTCCAGCCTGGGTAACAAGAGCGAAACTCCGTCTCAAAAAA
>sf45 count=357 pvalue=3e-119 (parent_pvalue=6e-158) parent=2 muts=
160:a/g Linup-refined
GGCCGGGCGCGGTGGCTCACGCCTGTAATCCCAGCACTTTGGGAGGCCGAGGCGGGAGGATCGCTTGAGCCCA
GGAGTTCGAGACCAGCCTGGGCAACATAGCGAGACCCCGTCTCTACAAAAAATATAAAAAATTAGCCGGGCGT
GGTGGCGCGCGCCTGTGGTCCCAGCTACTCGGGAGGCTGAGGCGGGAGGATCGCTTGAGCCCGGGAGGTCGAG
GCTGCAGTGAGCTGTGATCGCGCCACTGCACTCCAGCCTGGGCGACAGAGCGAGACCCTGTCTCAAAAAA
>sf46 count=149 pvalue=3e-104 (parent_pvalue=6e-4269) parent=1 muts=
266:a/c Linup-refined
GGCCGGGCGCGGTGGCTCAAGCCTGTAATCCCAGCACTTTGGGAGGCCGAGGCGGGTGGATCACGAGGTCAAG
AGATCGAGACCATCCTGGTCAACATGGTGAAACCCCGTCTCTACTAAAAAATATACAAAAAATTAGCTGGGCA
TGGTGGCGCGTGCCTGTAATCCCAGCTACTCAGGAGGCTGAGGCAGGAGAATTGCCTGAACCCAGGAGGCGGA
GGTTGCGGTGAGCCGAGATCGCGCCATTGCACTCCAGCCTGGGTAACAAGCGCGAAACTCCGTCTCAAAAAA

>sf47 count=369 pvalue=5e-92 (parent_pvalue=6e-4269) parent=1 muts=
265:g/a Linup-refined
GGCCGGGCGCGGTGGCTCANGCCTGTAATCCCAGCACTTTGGGAGGCCGAGGCGGGTGGATCACGAGGTCAAG
AGATCGAGACCATCCTGGTCAACATGGTGAAACCCCGTCTCTACTAAAAATACAAAAAATTAGCTGGGCATGG
TGGCGCGTGCCTGTAATCCCAGCTACTCAGGAGGCTGAGGCAGGAGAATTGCCTGAACCCAGGAGGCGGAGGT
TGCGGTGAGCCGAGATCGCGCCATTGCACTCCAGCCTGGGTAACAAAAGCGAAACTCCGTCTCAAAAAA
>sf48 count=63 pvalue=4e-80 (parent_pvalue=2e-48) parent=8 muts=
244:g/t Linup-refined
GGCCGGGCGCGGTGGCTCACGCCTGTAATCCTAGCACTTTGGGAGGCCGAGGCGGGTGGATCACCTGAGGTCA
GGAGTTCGAGACCAGCCTGGCCAACATGGCGAAACCCCGTCTCTACTAAAAATACAAAAATTAGCCGGGCGTG
GTGGCGGGCGCCTGTAATCCCAGCTACTCGGGAGGCTGAGGCAGGAGAATCGCTTGAACCCGGGGGGCGGAGG
TTGCAGTGAGCCGAGATCGCGCCACTTCACTCCAGCCTGGGCGAAAGAGCGAAACTCCGTCTCAAAAAA
>sf50 count=856 pvalue=4e-65 (parent_pvalue=5e-2242) parent=0 muts=
188:g/t Linup-refined
GGCCGGGCGCGGTGGCTCACGCCTGTAATCCCAGCACTTTGGGAGGCCGAGGCGGGCGGATCACTTGAGGTCA
GGAGTTCGAGACCAGCCTGGCCAACATGGCGAAACCCCGTCTCTACTAAAAATACAAAAATTAGCCGGGCGTG
GTGGCGCGCGCCTGTAATCCCAGCTACTCGGGAGGCTGAGGCATGAGAATCGCTTGAACCCGGGAGGCGGAGG
TTGCAGTGAGCCGAGATCGCGCCACTGCACTCCAGCCTGGGCGACAGAGCGAGACTCTGTCTCAAAAAA
>sf51 count=249 pvalue=2e-49 (parent_pvalue=6e-4269) parent=1 muts=
95:c/a Linup-refined
GGCCGGGCGCGGTGGCTCAAGCCTGTAATCCCAGCACTTTGGGAGGCCGAGGCGGGTGGATCACGAGGTCAAG
AGATCGAGACCATCCTGGTCAAAATGGTGAAACCCCGTCTCTACTAAAGATACAAAAAATTAGCTGGGCATGG
TGGCGCGTGCCTGTAATCCCAGCTACTCGGGAGGCTGAGGCAGGAGAATTGCCTGAACCCAGGAGGCGGAGGT
TGCGGTGAGCCGAGATCGCGCCATTGCACTCCAGCCTGGGTAACAAGAGCGAAACTCCGTCTCAAAAAA
>sf52 count=133 pvalue=4e-50 (parent_pvalue=6e-4269) parent=1 muts=
262:c/a Linup-refined
GGCCGGGCGCGGTGGCTCAAGCCTGTAATCCCAGCACTTTGGGAGGCCGAGGCGGGTGGATCACGAGGTCAAG
AGATCGAGACCATCCTGGTCAACATGGTGAAACCCCGTCTCTACTAAAAATACAAAAAATTAGCTGGGCATGG
TGGCGCGTGCCTGTAATCCCAGCTACTCAGGAGGCTGAGGCAGGAGAATTGCCTGAACCCAGGAGGCGGAGGT
TGCGGTGAGCCGAGATCGCGCCATTGCACTCCAGCCTGGGTAAAAAGAGCGAAACTCCGTCTCAAAAAA
>sf53 count=139 pvalue=2e-44 (parent_pvalue=6e-4269) parent=1 muts=
202:a/t Linup-refined
GGCCGGGCGCGGTGGCTCACGCCTGTAATCCCAGCACTTTGGGAGGCCGAGGCGGGTGGATCACGAGGTCAAG
AGATCGAGACCATCCTGGTCAACATGGTGAAACCCCGTCTCTACTAAAAATACAAAAAATTAGCTGGGCATGG
TGGCGCGTGCCTGTAATCCCAGCTACTCGGGAGGCTGAGGCAGGAGAATTGCCTGATCCCAGGAGGCGGAGGT
TGCGGTGAGCCGAGATCGCGCCATTGCACTCCAGCCTGGGTAACAAGAGCGAAACTCCGTCTCAAAAAA
>sf54 count=220 pvalue=1e-42 (parent_pvalue=4e-6183) parent=3 muts=
258:g/c Linup-refined
GGCCGGGCGCGGTGGCTCACGCCTGTAATCCCAGCACTTTGGGAGGCCGAGGCGGGCGGATCACGAGGTCAAG
AGATCGAGACCATCCTGGCCAACATGGTGAAACCCCGTCTCTACTAAAAATACAAAAATTAGCTGGGCGTGGT
GGCGCGCGCCTGTAGTCCCAGCTACTCGGGAGGCTGAGGCAGGAGAATCGCTTGAACCCGGGAGGCGGAGGTT
GCAGTGAGCCGAGATCGCGCCACTGCACTCCAGCCTGGCCGACAGAGCGAGACTCCGTCTCAAAAAA
>sf55 count=135 pvalue=1e-39 (parent_pvalue=2e-300) parent=17 muts=
194:t/c Linup-refined
GGCCGGGCGCGGTGGCTCACGCCTGTAATCCCAGCACTTTGGGAGGCCGAGGCGGGTGGATCACGAGGTCAGG
AGTTCAAGACCAGCCTGGCCAAGATGGTGAAACCCCGTCTCTACTAAAAATACAAAAATTAGCCGGGCGTGGT
GGCGGGCGCCTGTAATCCCAGCTACTCGGGAGGCTGAGGCAGAGAACTGCTTGAACCCGGGAGGCGGAGGTTG
CAGTGAGCCGAGATCGCGCCACTGCACTCCAGCCTGGGCGACAGAGCGAGACTCCGTCTCAAAAAA
>sf56 count=136 pvalue=1e-37 (parent_pvalue=1e-5) parent=9 muts=
177:a/g Linup-refined
AGCCGGGCGCGGTGGCTCACGCCTGTAATCCCAGCACTTTGGGAGGCCGAGGCGGGCGGATCGCTTGAGCCCA
GGAGTTCGAGACCAGCCTGGGCAACATGGCGAAACCCCGTCTCTACAAAAAATACAAAAATTAGCCGGGCGTG
GTGGCGCGCGCCTGTAGTCCCAGCTACTTGGGGGGCTGAGGTGGGAGGATCGCTTGAGCCCGGGAGGTCGAGG
CTGCAGTGAGCCGTGATCGCGCCACTGCACTCCAGCCTGGGCGACAGAGCGAGACCCTGTCTCAAAAAA

>sf57 count=112 pvalue=9e-33 (parent_pvalue=6e-158) parent=2 muts=
123:t/a Linup-refined
GGCCGGGCGCGGTGGCTCACGCCTGTAATCCCAGCACTTTGGGAGGCCGAGGCGGGAGGATCGCTTGAGCCCA
GGAGTTCGAGACCAGCCTGGGCAACATAGCGAGACCCCGTCTCTACAAAAAAAAAAAAATTAGCCGGGCGTGG
TGGCGCGCGCCTGTAGTCCCAGCTACTCGGGAGGCTGAGGCGGGAGGATCGCTTGAGCCCGGGAGGTCGAGGC
TGCAGTGAGCTGTGATCGCGCCACTGCACTCCAGCCTGGGCGACAGAGCGAGACCCTGTCTCAAAAAA
>sf58 count=93 pvalue=4e-30 (parent_pvalue=6e-158) parent=2 muts=
62:a/t Linup-refined
GGCCGGGCGCGGTGGCTCACGCCTGTAATCCCAGCACTTTGGGAGGCCGAGGCGGGAGGATGCTCTTGAGCCC
AGGAGTTCGAGACCAGCCTGGGCAACATAGCGAGACCCCGTCTCTACAAAAAATATAAAAAATTAGCCGGGCG
TGGTGGCGCGCGCCTGTAGTCCCAGCTACTCGGGAGGCTGAGGCGGGAGGATCGCTTGAGCCCGGGAGGTCGA
GGCTGCAGTGAGCTGTGATCGCGCCACTGCACTCCAGCCTGGGCGACAGAGCGAGACCCTGTCTCAAAAAA
>sf59 count=577 pvalue=4e-30 (parent_pvalue=5e-2242) parent=0 muts=
151:c/a Linup-refined
GGCCGGGCGCGGTGGCTCACGCCTGTAATCCCAGCACTTTGGGAGGCCGAGGCGGGCGGATCACCTGAGGTCA
GGAGTTCGAGACCAGCCTGGCCAACATGGTGAAACCCCGTCTCTACTAAAAATACAAAAATTAGCCGGGCGTG
GTGGCGAGCGCCTGTAATCCCAGCTACTCGGGAGGCTGAGGCAGGAGAATCGCTTGAACCCGGGAGGCGGAGG
TTGCAGTGAGCCGAGATCGCGCCACTGCACTCCAGCCTGGGCGACAGAGCGAGACTCCGTCTCAAAAAA
>sf60 count=103 pvalue=6e-30 (parent_pvalue=6e-4269) parent=1 muts=
262:c/g Linup-refined
GGCCGGGCGCGGTGGCTCACGCCTGTAATCCCAGCACTTTGGGAGGCCGAGGCGGGTGGATCACGAGGTCAAG
AGATCGAGACCATCCTGGTCAACATGGTGAAACCCCGTCTCTACTAAAAATACAAAAAATTAGCTGGGCATGG
TGGCGCGTGCCTGTAATCCCAGCTACTCGGGAGGCTGAGGCAGGAGAATTGCCTGAACCCAGGAGGCGGAGGT
TGCGGTGAGCCGAGATCGCGCCATTGCACTCCAGCCTGGGTAAGAAGAGCGAAACTCCGTCTCAAAAAA
>sf61 count=64 pvalue=8e-30 (parent_pvalue=3e-1473) parent=10 muts=
180:c/g Linup-refined
GGCCGGGCGCGGTGGCTCACGCCTATAATCCCAGCACTTTGGGAGGCCGAGGCGGGTGGATCACGAGGTCAAG
AGATCGAGACCATCCTGGTCAACGAGGTGAAACCCCGTCTCTACTAAAAATACAAAAATTAGCTGGGCATGGT
GGCGCGTGCCTGTAATCCCAGCTACTCGGGAGGGTGAGGCAGGAGAATTGCTTGAACCCAGAAGGCGGAGGTT
GCGGTGAGCCGAGATCGTGCCATTGCACTCCAGTCTGGGTAACAACAGCGAAACTCCGTCTCAAAAAA
>sf62 count=149 pvalue=9e-28 (parent_pvalue=6e-4269) parent=1 muts=
221:c/a Linup-refined
GGCCGGGCGCGGTGGCTCAAGCCTGTAATCCCAGCACTTTGGGAGGCCGAGGCGGGTGGATCACGAGGTCAAG
AGATCGAGACCATCCTGGTCAACATGGTGAAACCCCGTCTCTACTAAAAATACAAAAAATTAGCTGGGCATGG
TGGCGCGTGCCTGTAATCCCAGCTACTCAGGAGGCTGAGGCAGGAGAATTGCCTGAACCCAGGAGGCGGAGGT
TGAGGTGAGCCGAGATCGCGCCATTGCACTCCAGCCTGGGTAACAAGAGCGAAACTCCGTCTCAAAAAA
>sf63 count=315 pvalue=8e-25 (parent_pvalue=6e-4269) parent=1 muts=
278:t/c Linup-refined
GGCCGGGCGCGGTGGCTCANGCCTGTAATCCCAGCACTTTGGGAGGCCGAGGCGGGTGGATCACGAGGTCAAG
AGATCGAGACCATCCTGGTCAACATGGTGAAACCCCGTCTCTACTAAAAATACAAAAAATTAGCTGGGCATGG
TGGCGCGTGCCTGTAATCCCAGCTACTCAGGAGGCTGAGGCAGGAGAATTGCCTGAACCCAGGAGGCGGAGGT
TGCGGTGAGCCGAGATCGCGCCATTGCACTCCAGCCTGGGTAACAAGAGCGAAACTCCGCCTCAAAAAA
>sf64 count=155 pvalue=8e-22 (parent_pvalue=4e-6183) parent=3 muts=
195:t/a Linup-refined
GGCCGGGCGCGGTGGCTCACGCCTGTAATCCCAGCACTTTGGGAGGCCGAGGCGGGCGGATCACGAGGTCAAG
AGATCGAGACCATCCTGGCCAACATGGTGAAACCCCGTCTCTACTAAAAATACAAAAATTAGCCGGGCGTGGT
GGCGCGCGCCTGTAGTCCCAGCTACTCGGGAGGCTGAGGCAGGAGAATAGCTTGAACCCGGGAGGCGGAGGTT
GCAGTGAGCCGAGATCGCGCCACTGCACTCCAGCCTGGCGACAGAGCGAGACTCCGTCTCAAAAAA
>sf65 count=89 pvalue=8e-21 (parent_pvalue=6e-4269) parent=1 muts=
219:t/a Linup-refined
GGCCGGGCGCGGTGGCTCAAGCCTGTAATCCCAGCACTTTGGGAGGCCGAGGCGGGTGGATCACGAGGTCAAG
AGATCGAGACCATCCTGGTCAACATGGTGAAACCCCGTCTCTACTAAAAATACAAAAAATTAGCTGGGCGTGG
TGGCGCGCGCCTGTAATCCCAGCTACTCGGGAGGCTGAGGCAGGAGAATTGCCTGAACCCAGGAGGCGGAGGT
AGCGGTGAGCCGAGATCGCGCCATTGCACTCCAGCCTGGGTAACGAGAGCGAAACTCCGTCTCAAAAAA

>sf66 count=103 pvalue=9e-21 (parent_pvalue=6e-4269) parent=1 muts=
96:a/t Linup-refined
AGCCGGGCGCGGTGGCTCAAGCCTGTAATCCCAGCACTTTGGGAGGCCGAGGCGGGTGGATCACGAGGTCAAG
AGATCGAGACCATCCTGGTCAACTTGGTGAAACCCCGTCTCTACTAAAAATACAAAAAATTAGCTGGGCATGG
TGGCGCGTGCCTGTAATCCCAGCTACTCAGGAGGCTGAGGCAGGAGAATTGCCTGAACCCAGGAGGCGGAGGT
TGCGGTGAGCCGAGATCGCGCCATTGCACTCCAGCCTGGGTAACAAGAGCGAAACTCCGTCTCAAAAAA
>sf67 count=91 pvalue=3e-20 (parent_pvalue=6e-158) parent=6 muts=
67:g/t Linup-refined
GGCCGGGCGCGGTGGCTCACGCCTGTAATCCCAGCACTTTGGGAGGCCGAGGCGGGAGGATCGCTTGAGTCCA
GGAGTTCGAGACCAGCCTGGGCAACATAGCGAGACCCCGTCTCTACAAAAAATATAAAAAATTAGCCGGGCGT
GGTGGCGCGTGCCTGTAGTCCCAGCTACTCGGGAGGCTGAGGCGGGAGGATCGCTTGAGCCCAGGAGTTCGAG
GCTGCAGTGAGCTATGATCGCGCCACTGCACTCCAGCCTGGGCGACAGAGCGAGACCCTGTCTCAAAAAA
>sf68 count=252 pvalue=9e-20 (parent_pvalue=6e-158) parent=2 muts=
107:c/t Linup-refined
GGCCGGGCGCGGTGGCTCACGCCTGTAATCCCAGCACTTTGGGAGGCCGAGGCGGGAGGATCGCTTGAGCCCA
GGAGTTCGAGACCAGCCTGGGCAACATAGCGAGACCTCGTCTCTACAAAAAATATAAAAAATTAGCCGGGCGT
GGTGGCGCGCGCCTGTAGTCCCAGCTACTCGGGAGGCTGAGGTGGGAGGATCGCTTGAGCCCGGGAGGTCGAG
GCTGCAGTGAGCTGTGATCGCGCCACTGCACTCCAGCCTGGGCGACAGAGCGAGACCCTGTCTCAAAAAA
>sf69 count=92 pvalue=2e-18 (parent_pvalue=3e-1473) parent=10 muts=
106:c/a Linup-refined
GGCCGGGCGCGGTGGCTCACGCCTATAATCCCAGCACTTTGGGAGGCCGAGGCGGGTGGATCACGAGGTCAAG
AGATCGAGACCATCCTGGTCAACAAGGTGAAACACCGTCTCTACTAAAAATACAAAAATTAGCTGGGCATGGT
GGCGCGCGCCTGTAGTCCCAGCTACTCGGGAGGCTGAGGCAGGAGAATTGCTTGAACCCAGGAGGCGGAGGTT
GCGGTGAGCCGAGATCGCGCCATTGCACTCCAGCCTGGGTAACAAGAGCGAAACTCCGTCTCAAAAAA
>sf70 count=186 pvalue=1e-12 (parent_pvalue=6e-158) parent=6 muts=
33:g/a Linup-refined
GGCCGGGCGCGGTGGCTCACGCCTGTAATCCCAACACTTTGGGAGGCCGAGGCGGGAGGATCGCTTGAGGCCA
GGAGTTCGAGACCAGCCTGGGCAACATAGCGAGACCCCGTCTCTACAAAAAATATAAAAAATTAGCCGGGCGT
GGTGGCGCGCGCCTGTAGTCCCAGCTACTCGGGAGGCTGAGGCGGGAGGATCGCTTGAGCCCAGGAGTTCGAG
GCTGCAGTGAGCTATGATCGCGCCACTGCACTCCAGCCTGGGCGACAGAGCGAGACCCTGTCTCAAAAAA
>sf71 count=66 pvalue=6e-12 (parent_pvalue=6e-4269) parent=1 muts=
94:a/c Linup-refined
GGCCGGGCGCGGTGGCTCAAGCCTGTAATCCCAGCACTTTGGGAGGCCGAGGCGGGTGGATCACGAGGTCAAG
AGATCGAGACCATCCTGGTCACCATGGTGAAACCCCGTCTCTACTAAAAATACAAAAAATTAGCTGGGCATGG
TGGCGCGTGCCTGTAATCCCAGCTACTCAGGAGGCTGAGGCAGGAGAATTGCCTGAACCCAGGAGGCGGAGGT
TGCGGTGAGCCGAGATCGCGCCATTGCACTCCAGCCTGGGTAACAAGAGCGAAACTCCGTCTCAAAAAA
>sf72 count=78 pvalue=1e-11 (parent_pvalue=6e-1473) parent=15 muts=
222:a/c Linup-refined
GGCCGGGCGCGGTGGCTCACGCCTGTAATCCCAGCACTTTGGGAGGCCGAGGCGGGCGGATCACCTGAGGTCG
GGAGTTCGAGACCAGCCTGACCAACATGGAGAAACCCCGTCTCTACTAAAAATACAAAATTAGCCGGGCGTGG
TGGCGCATGCCTGTAATCCCAGCTACTCGGGAGGCTGAGGCAGGAGAATCGCTTGAACCCGGGAGGCGGAGGT
TGCCGTGAGCCGAGATCGCGCCATTGCACTCCAGCCTGGGCAACAAGAGCGAAACTCCGTCTCAAAAAA
>sf73 count=118 pvalue=1e-11 (parent_pvalue=6e-4269) parent=1 muts=
132:t/a Linup-refined
GGCCGGGCGCGGTGGCTCACGCCTGTAATCCCAGCACTTTGGGAGGCCGAGGCGGGTGGATCACGAGGTCAAG
AGATCGAGACCATCCTGGTCAACATGGTGAAACCCCGTCTCTACTAAAAATACAAAAAAATAGCTGGGCATGG
TGGCGCGTGCCTGTAATCCCAGCTACTCAGGAGGCTGAGGCAGGAGAATTGCCTGAACCCAGGAGGCGGAGGT
TGCGGTGAGCCGAGATCGCGCCATTGCACTCCAGCCTGGGTAACAAGAGCGAAACTCCGTCTCAAAAAA
>sf74 count=316 pvalue=4e-11 (parent_pvalue=4e-6183) parent=3 muts=
233:a/g Linup-refined
GGCCGGGCGCGGTGGCTCACGCCTGTAATCCCAGCACTTTGGGAGGCCGAGGCGGGCGGATCACGAGGTCAAG
AGATCGAGACCATCCTGGCCAACATGGTGAAACCCCGTCTCTACTAAAAATACAAAAAATTAGCTGGGCGTGG
TGGCGCGCGCCTGTAGTCCCAGCTACTCGGGAGGCTGAGGCAGGAGAATCGCTTGAACCCGGGAGGCGGAGGT

TGCAGTGAGCCGAGGTCGCGCCACTGCACTCCAGCCTGGCGCCTGGTGACGGAGCGAGACTCTGTCTCAAAAA
A
>sf75 count=209 pvalue=7e-11 (parent_pvalue=5e-2242) parent=0 muts=
161:a/t Linup-refined
GGCCGGGCGCGGTGGCTCACGCCTGTAATCCCAGCACTTTGGGAGGCCGAGGCGGGCGGATCACCTGAGGTCA
GGAGTTCGAGACCAGCCTGGCCAACATGGTGAAACCCCGTCTCTACTAAAAATACAAAAATTAGCCGGGCGTG
GTGGCGCGCGCCTGTATTCCCAGCTACTCGGGAGGCTGAGGCAGGAGAATCGCTTGAACCCGGGAGGCGGAGG
TTGCAGTGAGCCGAGATCGCGCCACTGCACTCCAGCCTGGGCGACAGAGCGAGACTCCGTCTCAAAAAA
>sf76 count=112 pvalue=9e-11 (parent_pvalue=6e-4269) parent=1 muts=
186:c/g Linup-refined
GGCCGGGCGCGGTGGCTCACGCCTGTAATCCCAGCACTTTGGGAGGCCGAGGCGGGTGGATCACGAGGTCAAG
AGATCGAGACCATCCTGGTCAACATGGTGAAACCCCGTCTCTACTAAAAATACAAAAAATTAGCTGGGCATGG
TGGCGCGTGCCTGTAATCCCAGCTACTCAGGAGGCTGAGGGAGGAGAATTGCCTGAACCCAGGAGGCGGAGGT
TGCGGTGAGCCGAGATCGCGCCATTGCACTCCAGCCTGGGTAACAAGAGCGAAACTCCGTCTCAAAAAA
>sf77 count=155 pvalue=1e-10 (parent_pvalue=5e-2242) parent=0 muts=
161:a/c Linup-refined
AGCCGGGCGCGGTGGCTCACGCCTGTAATCCCAGCACTTTGGGAGGCCGAGGCGGGCGGATCACTTGAGGTCA
GGAGTTCGAGACCAGCCTGGCCAACATGGTGAAACCCCGTCTCTACTAAAAATACAAAAATTAGCCGGGCGTG
GTGGCGCGCGCCTGTACTCCCAGCTACTCGGGAGGCTGAGGCAGGAGAATCGCTTGAACCCGGGAGGCGGAGG
TTGCAGTGAGCCGAGATCGCGCCACTGCACTCCAGCCTGGGCGACAGAGCGAGACTCCGTCTCAAAAAA
>sf78 count=63 pvalue=3e-10 (parent_pvalue=1e-5) parent=9 muts= 197:c/a
Linup-refined
GGCCGGGCGCGGTGGCTCACGCCTGTAATCCCAGCACTTTGGGAGGCCGAGGCGGGCGGATCGCTTGAGCCCA
GGAGTTCGAGACCAGCCTGGGCAACATGGCGAAACCCCGTCTCTACAAAAAATACAAAAATTAGCCGGGCGTG
GTGGCGCGCGCCTGTAGTCCCAGCTACTCGGGAGGCTGAGGTGGGAGGATCGATTGAGCCCGGGAGGTCGAGG
CTGCAGTGAGCCGTGATCGCGCCACTGCACTCCAGCCTGGGCGACAGAGCGAGACCCTGTCTCAAAAAA
>sf79 count=68 pvalue=9e-9 (parent_pvalue=6e-158) parent=6 muts= 60:t/c
Linup-refined
GGCCGGGCGCGGTGGCTCACGCCTGTAATCCCAGCACTTTGGGAGGCCGAGGCGGGAGGACCGCTTGAGGCCA
GGAGTTCGAGACCAGCCTGGGCAACATAGCGAGACCCCGTCTCTACAAAAAATNTAAAAAATTAGCCGGGCGT
GGTGGCGCGCGCCTGTAGTCCCAGCTACTCGGGAGGCTGAGGCGGGAGGATCGCTTGAGCCCAGGAGTTCGAG
GCTGCAGTGAGCTATGATTGCGCCACTGCACTCCAGCCTGGGCGACAGAGCGAGACCCTGTCTCAAAAAA
>sf80 count=277 pvalue=2e-8 (parent_pvalue=9e-1230) parent=13 muts=
234:t/c Linup-refined
GGCCGGGCGCGGTGGCTCACGCCTGTAATCCCAGCACTTTGGGAGGCCGAGGCGGGCGGATCACCTGAGGTCA
GGAGTTCGAGACCAGCCTGGCCAACATGGTGAAACCCCGTCTCTACTAAAAATACAAAAATTAGCCGGGCGTG
GTGGCGGGCGCCTGTAATCCCAGCTACTCGGGAGGCTGAGGCAGGAGAATCGCTTGAACCCGGGAGGCGGAGG
TTGCAGTGAGCCGAGACCGCGCCATTGCACTCCAGCCTGGGCGACAAGAGCGAAACTCCGTCTCAAAAAA
>sf81 count=157 pvalue=4e-8 (parent_pvalue=4e-6183) parent=3 muts=
76:t/c Linup-refined
GGCCGGGCGCGGTGGCTCACGCCTGTAATCCCAGCACTTTGGGAGGCCGAGGCGGGCGGATCACGAGGTCAAG
AGACCGAGACCATCCTGGCCAACATGGTGAAACCCCGTCTCTACTAAAAATACAAAAATTAGCTGGGCGTGGT
GGCGCGCGCCTGTAGTCCCAGCTACTCGGGAGGCTGAGGCAGGAGAATCGCTTGAACCCGGGAGGCGGAGGTT
GCAGTGAGCCGAGATCGCGCCACTGCACTCCAGCCTGGGCGACAGAGCGAGACTCCGTCTCAAAAAA
>sf82 count=264 pvalue=8e-8 (parent_pvalue=6e-4269) parent=1 muts=
281:c/a Linup-refined
GGCCGGGCGCGGTGGCTCAAGCCTGTAATCCCAGCACTTTGGGAGGCCGAGGCGGGTGGATCACGAGGTCAAG
AGATCGAGACCATCCTGGTCAACATGGTGAAACCCCGTCTCTACTAAAAATACAAAAAATTAGCTGGGCATGG
TGGCGCGTGCCTGTAATCCCAGCTACTCAGGAGGCTGAGGCAGGAGAATTGCCTGAACCCAGGAGGCGGAGGT
TGCGGTGAGCCGAGATCGCGCCATTGCACTCCAGCCTGGGTAACAAGAGCGAAACTCCGTCTAAAAAAA
>sf83 count=56 pvalue=1e-7 (parent_pvalue=6e-158) parent=6 muts=
196:g/c Linup-refined
GGCTGGGCGCGGTGGCTCACGCCTGTAATCCCAGCACTTTGGGAGGCCGAGGCGGGAGGATCGCTTGAGGCCA
GGAGTTCGAGACCAGCCTGGGCAACATAGCGAGACCCCGTCTCTACAAAAAATATAAAAAATTAGCCGGGCGT

GGTGGCGCGTGCCTGTAGTCCCAGCTACTCGGGAGGCTGAGGCGGGAGGATCCCTTGAGCCCAGGAGTTCGAG
GCTGCAGTGAGCTATGATCGCGCCACTGCACTCCAGCCTGGGCGACAGAGCGAGACCCTGTCTCAAAAAA
>sf84 count=118 pvalue=5e-7 (parent_pvalue=4e-6183) parent=3 muts=
70:a/c Linup-refined
GGCCGGGCGCGGTGGCTCACGCCTGTAATCCCAGCACTTTGGGAGGCCGAGGCGGGCGGATCACGAGGTCCAG
AGATCGAGACCANCCTGGCCAACATGGCGAAACCCCGTCTCTACTAAAAATACAAAAATTAGCCGGGCGTGGT
GGCGCGCGCCTGTAGTCCCAGCTACTCGGGAGGCTGAGGCGGGAGAATCGCTTGAACCCGGGAGGCGGAGGTT
GCAGTGAGCCGAGATCGCGCCACTGCACTCCAGCCTGGGCGACAAGAGCGAGACTCTGTCTCAAAAAA
>sf85 count=68 pvalue=1e-6 (parent_pvalue=6e-4269) parent=1 muts=
74:g/c Linup-refined
GGCCGGGCGCGGTGGCTCANGCCTGTAATCCCAGCACTTTGGGAGGCCGAGGCGGGTGGATCACGAGGTCAAG
ACATCGAGACCATCCTGGTCAACATGGTGAAACCCCGTCTCTACTAAAAATACAAAAAATTAGCTGGGCATGG
TGGCGCGTGCCTGTAATCCCAGCTACTCAGGAGGCTGAGGCAGGAGAATTGCCTGAACCCAGGAGGCGGAGGT
TGCGGTGAGCCGAGATCGCGCCATTGCACTCCAGCCTGGGTAACAAGAGCGAAACTCCGTCTCAAAAAA
>sf86 count=125 pvalue=3e-6 (parent_pvalue=6e-4269) parent=1 muts=
229:c/g Linup-refined
GGCCGGGCGCGGTGGCTCANGCCTGTAATCCCAGCACTTTGGGAGGCCGAGGCGGGTGGATCACGAGGTCAAG
AGATCGAGACCATCCTGGTCAACATGGTGAAACCCCGTCTCTACTAAAAATACAAAAAATTAGCTGGGCATGG
TGGCGCGTGCCTGTAATCCCAGCTACTCAGGAGGCTGAGGCAGGAGAATTGCCTGAACCCAGGAGGCGGAGGT
TGCGGTGAGCGGAGATCGCGCCATTGCACTCCAGCCTGGGTAACAAGAGCGAAACTCCGTCTCAAAAAA
>sf87 count=110 pvalue=4e-6 (parent_pvalue=1e-5) parent=9 muts= 195:c/g
Linup-refined
GGCCGGGCGCGGTGGCTCACGCCTGTAATCCCAGCACTTTGGGAGGCCGAGGCGGGCGGATCGCTTGAGCCCA
GGAGTTCGAGACCAGCCTGGGCAACATGGCGAAACCCCGTCTCTACAAAAAATACAAAAATTAGCCGGGCGTG
GTGGCGCGCGCCTGTAGTCCCAGCTACTCGGGAGGCTGAGGTGGGAGGATGGCTTGAGCCCGGGAGGTCGAGG
CTGCAGTGAGCCGTGATCGCGCCACTGCACTCCAGCCTGGGCGACAGAGCGAGACCCTGTCTCAAAAAA
>sf88 count=90 pvalue=7e-7 (parent_pvalue=1e-5) parent=9 muts= 92:c/a
Linup-refined
GGCCGGGCGCGGTGGCTCACGCCTGTAATCCCAGCACTTTGGGAGGCCGAGGCGGGCGGATCGCTTGAGCCCA
GGAGTTCGAGACCAGCCTGGGAAACATGGCGAAACCCCGTCTCTACAAAAAATACAAAAAATTAGCCGGGCGT
GGTGGCGCGCGCCTGTAGTCCCAGCTACTCGGGAGGCTGAGGTGGGAGGATCGCTTGAGCCCGGGAGGTCGAG
GCTGCAGTGAGCCGTGATCGCGCCACTGCACTCCAGCCTGGGCGACAGAGCGAGACCCTGTCTCAAAAAA
>sf89 count=50 pvalue=1e-5 (parent_pvalue=6e-158) parent=2 muts=
100:t/g Linup-refined
GGCCGGGCGCGGTGGCTCACGCCTGTAATCCCAGCACTTTGGGAGGCCGAGGCGGGAGGATCGCTTGAGCCCA
GGAGTTCGAGACCAGCCTGGGCAACATAGGGAGACCCCGTCTCTACAAAAAATAAAAAAATTAGCCGGGCGTG
GTGGCGCGCGCCTGTAGTCCCAGCTACTCGGGAGGCTGAGGTGGGAGGATCGCTTGAGCCCGGGAGGTCGAGG
CTGCAGTGAGCTGTGATCGCGCCACTGCACTCCAGCCTGGGCGACAGAGTGAGACCCTGTCTCAAAAAA
>sf90 count=72 pvalue=8e-5 (parent_pvalue=4e-6183) parent=3 muts=
71:a/c Linup-refined
GGCCGGGCGCGGTGGCTCACGCCTGTAATCCCAGCACTTTGGGAGGCCGAGGCGGGCGGATCACGAGGTCACG
AGATCGAGACCANCCTGGCCAACATGGTGAAACCCCGTCTCTACTAAAAATACAAAAATTAGCCGGGCGTGGT
GGCGCGCGCCTGTAGTCCCAGCTACTCGGGAGGCTGAGGCAGGAGAATCGCTTGAACCCGGGAGGCGGAGGTT
GCAGTGAGCCGAGATCGCGCCACTGCACTCCAGCCTGGGCGACAGAGCGAGACTCCGTCTCAAAAAA
>sf91 count=128 pvalue=1e-4 (parent_pvalue=8e-89) parent=16 muts=
266:a/t Linup-refined
GGCCGGGCGCGGTGGCTCACGCCTGTAATCCCAGCACTTTGGGAGGCCGAGGCGGGCGGATCACGAGGTCAGG
AGTTCGAGACCAGCCTGGCCAACATGGTGAAACCCCGTCTCTACTAAAAATACAAAAAATTAGCCGGGCGTGG
TGGCGGGCGCCTGTAATCCCAGCTACTCGGGAGGCTGAGGCAGGAGAATCGCTTGAACCCGGGAGGCGGAGGT
TGCAGTGAGCCGAGATCGCGCCACTGCACTCCAGCCCGGGCGACAGTGCGAGACTCCGTCTCAAAAAA
>sf92 count=92 pvalue=3e-4 (parent_pvalue=1e-5) parent=9 muts= 172:t/c
Linup-refined
GGCCGGGCGCGGTGGCTCACGCCTGTAATCCCAGCACTTTGGGAGGCCGAGGCGGGCGGATCGCTTGAGCCCA
GGAGTTCGAGACCAGCCTGGGCAACATGGCGAAACCCCGTCTCTACAAAAAATACAAAAAATTAGCCGGGCGT

GGTGGCGCGCGCCTGTAGTCCCAGCTACCCGGGAGGCTGAGGTGGGAGGATCGCCTGAGCCCGGGAGGTCGAG
GCTGCAGTGAGCCGTGATCGCGCCACTGCACTCCAGCCTGGGCGACAGAGCGAGACCCTGTCTCAAAAAA
>sf93 count=268 pvalue=4e-4 (parent_pvalue=5e-2242) parent=0 muts=
199:t/g Linup-refined
GGCCGGGCGCGGTGGCTCACGCCTGTAATCCCAGCACTTTGGGAGGCCGAGGCGGGCGGATCACCTGAGGTCA
GGAGTTCGAGACCAGCCTGGCCAACATGGTGAAACCCCGTCTCTACTAAAAATACAAAAATTAGCCGGGCGTG
GTGGCGCGCGCCTGTAATCCCAGCTACTCGGGAGGCTGAGGCAGGAGAATCGCTGGAACCCGGGAGGCGGAGG
TTGCAGTGAGCCGAGATCGCGCCACTGCACTCCAGCCTGGGCGACAGAGCGAGACTCCGTCTCAAAAAA
>AluJo #SINE/Alu @Primates [S:85] RepbaseID: ALU
ggccgggcgcggtggctcacgcctgtaatcccagcactttgggaggccgaggcgggaggatcgcttgagccca
ggagttcgagaccagcctgggcaacatagcgagaccccgtctctacaaaaaatacaaaaattagccgggcgtg
gtggcgcgcgcctgtagtcccagctactcgggaggctgaggcaggaggatcgcttgagcccaggagttcgagg
ctgcagtgagctatgatcgcgccactgcactccagcctgggcgacagagcgagaccctgtctcaaaaaa
>AluJr4 #SINE/Alu @Primates [S:85]
ggccgggcgcggtggctcacgcctgtaatcccagcactttgggaggccgaggcgggaggatcgcttgaggcca
ggagttcgagaccagcctgggcaacatagcgagaccccgtctctacaaaaaatntaaaaattagccgggcgtg
gtggcgcgcgcctgtagtcctagctactcgggaggctgaggcaggaggatcgcttgagcccaggagttcgagg
ttacagtgagctatgatcgcgccactgcactccagcctgggcgacagagcgagaccctgtctcaaaaaa
>AluSc #SINE/Alu @Primates [S:85]
ggccgggcgcggtggctcacgcctgtaatcccagcactttgggaggccgaggcgggcggatcacgaggtcaag
agatcgagaccatcctggccaacatggtgaaaccccgtctctactaaaaatacaaaaattagccgggcgtggt
ggcgcgcgcctgtagtcccagctactcgggaggctgaggcaggagaatcgcttgaacccgggaggcggaggtt
gcagtgagccgagatcgcgccactgcactccagcctggcgacagagcgagactccgtctcaaaaaa
>AluSc5 #SINE/Alu @Primates [S:85]
ggccgggcgcggtggctcacgcctgtaatcccagcactttgggaggccgaggcgggcggatcacgaggtcagg
agatcgagaccatcctggccaacatggtgaaaccccgtctctactaaaaatacaaaaattagctgggcgtggt
ggcgcgtgcctgtaatcccagctactcgggaggctgaggcaggagaatcgcttgaaccagggagtcggaggtt
gcagtgagccgagatcgcgccactgcactccagcctggcgacagagcgagactccgtctcaaaaaa
>AluSc8 #SINE/Alu @Primates [S:85]
ggccgggcgcggtggctcacgcctgtaatcccagcactttgggaggccgaggcgggcggatcacgaggtcagg
agatcgagaccatcctggctaacacggtgaaaccccgtctctactaaaaatacaaaaaattagccgggcgtgg
tggcacgcgcctgtagtcccagctactcgggaggctgaggcaggagaatcgcttgaacccgggaggcggaggt
tgcagtgagccgagatcgcgccactgcactccagcctgggcgacagagcgagactccgtctcaaaaaa
>AluSg #SINE/Alu @Primates [S:85]
ggccgggcgcggtggctcacgcctgtaatcccagcactttgggaggccgaggcgggcggatcacgaggtcagg
agttcgagaccagcctggccaacatggtgaaaccccgtctctactaaaaatacaaaaattagccgggcgtggt
ggcgcgcgcctgtaatcccagctactcgggaggctgaggcaggagaatcgcttgaacccgggaggcggaggtt
gcagtgagccgagatcgcgccactgcactccagcctgggcgacagagcgagactccgtctcaaaaaa
>AluSg4 #SINE/Alu @Primates [S:85]
ggccgggcgcggtggctcacgcctgtaatcccagcactttgggaggccgaggcgggtggatcacgaggtcagg
agttcgagaccagcctggccaagatggtgaaaccccgtctctactaaaaatacaaaaattagccgggcgcggt
ggcgggcgcctgtaatcccagctactcgggaggctgaggcaggagaatcgcttgaacccgggaggcggaggtt
gcagtgagccgagatcgcgccactgcactccagcctgggcgacagagcgagactccgtctcaaaaaa
>AluSg7 #SINE/Alu @Primates [S:85]
ggccgggcgcggtggctcacgcctgtaatcccagcactttgggaggccgaggcgggtggatcacgaggtcagg
agttcaagaccagcctggccaagatggtgaaaccccgtctctactaaaaatacaaaaattagccgggcgtggt
ggcgggcgcctgtaatcccagctactcgggaggctgaggcagagaattgcttgaacccgggaggcggaggttg
cagtgagccgagatcgcgccactgcactccagcctgggcgacagagcgagactccgtctcaaaaaa
>AluSp #SINE/Alu @Primates [S:85]
ggccgggcgcggtggctcacgcctgtaatcccagcactttgggaggccgaggcgggcggatcacctgaggtcg
ggagttcgagaccagcctgaccaacatggagaaaccccgtctctactaaaaatacaaaaattagccgggcgtg
gtggcgcgcgcctgtaatcccagctactcgggaggctgaggcaggagaatcgcttgaacccgggaggcggagg
ttgcggtgagccgagatcgcgccattgcactccagcctgggcaacaagagcgaaactccgtctcaaaaaa
>AluSq #SINE/Alu @Primates [S:85]

```
ggccgggcgcggtggctcacgcctgtaatcccagcactttgggaggccgaggcgggcggatcacctgaggtca
ggagttcgagaccagcctggccaacatggtgaaaccccgtctctactaaaaatacaaaaattagccgggcgtg
gtggcgggcgcctgtaatcccagctactcgggaggctgaggcaggagaatcgcttgaacccgggaggcggagg
ttgcagtgagccgagatcgcgccactgcactccagcctgggcaacaagagcgaaactccgtctcaaaaaa
>AluSq4 #SINE/Alu @Primates  [S:85]
ggccgggcgcggtggctcacgcctgtaatcccagcactttgggaggccgaggcgggcggatcactgaggtcag
gagttcgagaccagcctggccaacatggtgaaaccccgtctctactaaaaatacaaaaattagccgggcgtgg
tggcgggcgcctgtaatcccagctactcgggaggctgaggcaggagaatcgcttgaaaccggaaggcggaggt
tgcagtgagccgagatcgcgccactgcactccagcctgggcaacaagagcgaaactccgtctcaaaaaa
>AluSq10 #SINE/Alu @Primates  [S:85]
ggccgggcgcggtggctcacgcctgtaatcccagcactttgggaggccgaggcgggtggatcacctgaggtca
ggagttctagaccagcctggccaacatggtgaaaccccgtctctactaaaaatacaaaaattagccgggcgtg
gtggcaggcgcctgtaatcccagctactcgggggccgaggcaggagaatcgcttgaacccgggaggcggagg
ttgcagtgagccgagatcgcgccatcgcactccagcctgggggacaagagcgagacttcgtctcaaaaaa
>AluSx3 #SINE/Alu @Primates  [S:85]
ggccgggcgcggtggctcacgcctgtaatcccagcactttgggaggccgaggcgggcggatcacgaggtcagg
agttcgagaccagcctggccaacatggtgaaaccccgtctctactaaaaatacaaaaaattagccgggcgtgg
tggcgcgcgcctgtagtcccagctactcgggaggctgaggcaggagaatcgcttgaacccgggaggcggaggt
tgcagtgagccgagatcgcgccactgcactccagcctgggcgacagagcgagactccgtctcaaaaaa
>AluSx4 #SINE/Alu @Primates  [S:85]
ggccgggcgcggtggctcacgcctgtaatcccagcactttgggaggccgaggcgggcggatcactgaggtcag
gagttcgagaccagcctggccaatatggtgaaaccccgtctctactaaaaatacaaaaattagccgggcgtgg
tggcgcgcgcctgtagtcccagctactcgggaggctgaggcagaagaatcgcttgaacccgggaggcggaggt
tgcagtgagccgagatcgcgccactgcactccagcctgggcgacagagcgagactccgtctcaaaaaa
>AluSz #SINE/Alu @Primates  [S:85]
ggccgggcgcggtggctcacgcctgtaatcccagcactttgggaggccgaggcgggcggatcacttgaggtca
ggagttcgagaccagcctggccaacatggtgaaaccccgtctctactaaaaatacaaaaattagccgggcgtg
gtggcgcgcgcctgtaatcccagctactcgggaggctgaggcaggagaatcgcttgaacccgggaggcggagg
ttgcagtgagccgagatcgcgccactgcactccagcctgggcgacagagcgagactccgtctcaaaaaa
>AluSz6 #SINE/Alu @Primates  [S:85]
ggccgggcgcggtggctcacgcctgtaatcccagcactttgggaggccgaggcgggcggatcacttgaggcca
ggagttcgagaccagcctggccaacatggcaaaaccccgtctctactaaaaatacaaaaattagccgggcgtg
gtggcgcgcgcctgtaatcccagctactcgggaggctgaggcacgagaatcgcttgaacccgggaggcggagg
ttgcagtgagccgagatcgcgccactgcactccagcctgggcgacagagcgagactctgtctcaaaaaa
>AluTa7 #SINE/Alu @Platyrrhini  [S:40,50]
ggccgggcgcggtggctcacgcctgtaatcccagcactttgggaggccgaggcgggcggatcacgaggtcaag
agatcgagaccatcctggccaacatggtgaaaccccgtctctactaaaaatacaaaaaaattagctgggcgtg
gtggcgcgcgcctgtagtcccagctactcgggaggctgaggcaggagaattgcttgaacccgggaggcggagg
ttgcagtgagccgaggtcgcgccactgcactccagcctggcgcctggtgacggagtgagactctgtctcaaaa
aa
>AluTa10 #SINE/Alu @Platyrrhini  [S:40,50]
ggccgggcgcggtggctcacgcctrtaatcccagcactttgggaggccgaggcgggtggatcacgaggtcaag
agatcgagaccatcctggtcaacatggtgaaaccccgtctctactaaaaatacaaaaattagctgggcatggt
ggcgcgcgcctgtagtcccagctactcgggaggctgaggcaggagaattgcttgaacccaggaggcggaggtt
gcggtgagccgagatcgcgccattgcactccagcctgggtaacaagagcgaaactccgtctcaaaaaa
>AluY #SINE/Alu @Primates  [S:35,85]
ggccgggcgcggtggctcacgcctgtaatcccagcactttgggaggccgaggcgggcggatcacgaggtcagg
agatcgagaccatcctggctaacacggtgaaaccccgtctctactaaaaatacaaaaaattagccgggcgtgg
tggcgggcgcctgtagtcccagctactcgggaggctgaggcaggagaatggcgtgaacccgggaggcggagct
tgcagtgagccgagatcgcgccactgcactccagcctgggcgacagagcgagactccgtctcaaaaaa
>AluYa5 #SINE/Alu @Primates  [S:85]
ggccgggcgcggtggctcacgcctgtaatcccagcactttgggaggccgaggcgggcggatcacgaggtcagg
agatcgagaccatcccggctaaaacggtgaaaccccgtctctactaaaaatacaaaaaattagccgggcgtag
```

```
tggcgggcgcctgtagtcccagctacttgggaggctgaggcaggagaatggcgtgaacccgggaggcggagct
tgcagtgagccgagatcccgccactgcactccagcctgggcgacagagcgagactccgtctcaaaaaa
>AluYa8 #SINE/Alu @Primates  [S:85]
ggccgggcgcggtggctcacgcctgtaatcccagcactttgggaggccgaggcgggcggatcacgaggtcagg
agatcgagaccatcccggctaaaacggtgaaaccccgtctctactaaaactacaaaaaatagccgggcgtagt
ggcgggcgcctgtagtcctagctacttgggaggctgaggcaggagaatggcgtgaacccgggaggcggagctt
gcagtgagccgagatcccgccactgcactccagcctgggcgacagagcgagactccgtctcaaaaaa
>AluYb8 #SINE/Alu @Primates  [S:85]
ggccgggcgcggtggctcacgcctgtaatcccagcactttgggaggccgaggcgggtggatcatgaggtcagg
agatcgagaccatcctggctaacaaggtgaaaccccgtctctactaaaaatacaaaaaattagccgggcgcag
tggcgggcgcctgtagtcccagctactcgggaggctgaggcaggagaatggcgtgaacccgggaagcggagct
tgcagtgagccgagattgcgccactgcagtccgcagtccggcctgggcgacagagcgagactccgtctcaaaa
aa
```
