# Supplementary material for: Framework of the Alu Subfamily Evolution in the Platyrrhine Three-Family Clade of Cebidae, Callithrichidae, and Aotidae
Source: Genes (Basel). 2023 Jan 18;14(2):249. doi: 10.3390/genes14020249 (PMC9956951; doi:10.3390/genes14020249)
Supplement: Supplementary file 1 [file genes-14-00249-s001.zip › Supplementary File S2.pdf]

>subfamily0 count=28821 pvalue=5e-388 (parent_pvalue=5e-388)
ggccgggcgcggtggctcaagcctgtaatcccagcactttgggaggccgaggcgggtggatcacgaggtcaagagat
cgagaccatcctggtcaacatggtgaaaccccgtctctactaaaaatacaaaaaAttagctgggcatggtggcgcgt
gcctgtaatcccagctactcaggaggctgaggcaggagaattgcctgaacccaggaggcggaggttgcggtgagccg
agatcgcgccattgcactccagcctgggtaacAagagcgaaactccgtctc
>subfamily1 count=580 pvalue=8e-118 (parent_pvalue=5e-388) parent=0 muts=
199:c/t 162:a/g 19:a/c
ggccgggcgcggtggctcacgcctgtaatcccagcactttgggaggccgaggcgggtggatcacgaggtcaagagat
cgagaccatcctggtcaacatggtgaaaccccgtctctactaaaaatacaaaaattagctgggcatggtggcgcgtg
cctgtagtcccagctactcgggaggctgaggcaggagaattgcttgaacccaggaggcggaggttgcggtgagccga
gatcgcgccattgcactccagcctgggtaacAagagcgaaactccgtctc
>subfamily2 count=396 pvalue=7e-139 (parent_pvalue=5e-388) parent=0 muts=
186:g/a 99:t/a 66:g/a
ggccgggcgcggtggctcaagcctgtaatcccagcactttgggaggccgaggcgggtggatcacaaggtcaagagat
cgagaccatcctggtcaacaaggtgaaaccccgtctctactaaaaatacaaaaaAttagctgggcatggtggtgcgt
gcctgtaatcccagctactcaggaggctgagacaggagaattgcctgaacccaggaggcggaggttgcggtgagccg
agatcgcgccattgcactccagcctgggtaacAagagcgaaactccgtctc
>subfamily3 count=56 pvalue=4e-102 (parent_pvalue=5e-388) parent=0 muts=
87:t/g 77:a/t 65:-/t
ggccgggcgcggtggctcacgcctgtaatcccagcactttgggaggccgaggcgggtggatcacaaggtcaggagtt
cgagaccagcctggccaaaatgttgaaaccccgtctctactaaaaatacaaaaattagctgggcgcggtggcacgcg
cctgtaatcccagctactcaggaggctgaggcaggagaatcgcttgaacctgggaggcggaggttgcagtgagccga
gatcgtgccattgcactccagcctgggtgacagagtgagactctgtctc
>subfamily4 count=1544 pvalue=1e-641 (parent_pvalue=5e-388) parent=0 muts=
203:a/g 174:c/t
ggccgggcgcggtggctcaagcctgtaatcccagcactttgggaggccgaggcgggtggatcacgaggtcgagagat
cgagaccatcctggtcaacatggtgaaaccccgtctctactaaaaatacaaaaaActagctgggcgtggtggcgcgt
gcctgtaatcccagctacttaggaggctgaggcaggagaattgcctgagcccaggaggcggaggttgcggtgagccg
agatcgcgccattgcactccagcctgggtaacAagagcgaaactccgtctc
>subfamily5 count=1171 pvalue=1e-451 (parent_pvalue=5e-388) parent=0 muts=
159:g/a 87:t/a
ggccgggcgcggtggctcaagcctgtaatcccagcactttgggaggccgaggcgggtggatcacaaggtcgagagat
cgagaccaacctggtcaacatggtgaaaccccgtctctactaaaaatacaaaaaAttagctgggcatggtggcgcgt
gcctataatcccagctactcaggaggctgaggcaggagaattgcctgaacccaggaggcggaggttgcggtgagccg
agatcgcgccattgcactccagcctgggtaacAagagcgaaactccgtctc
>subfamily6 count=233 pvalue=3e-62 (parent_pvalue=8e-118) parent=1 muts=
212:g/a 51:g/a
ggccgggcgcggtggctcacgcctataatcccagcactttgggaggccgagacgggtggatcacgaggtcaagagat
cgagaccatcctggtcaacatggtgaaaccccgtctctactaaaaatacaaaaattagctgggcatggtggcgcacg
cctgtagtcccagctactcgggaggctgaggcaggagaattgcttgaacccaggagacggaggttgcggtgagccga
gatcgcgccattgcactccagcctgggtaacAagagcgaaactccgtctc
>subfamily7 count=79 pvalue=1e-51 (parent_pvalue=8e-118) parent=1 muts=
265:g/c 89:c/t
ggccgggcgcggtggctcacgcctataatcccagcactttgggaggccgaggcgggtggatcacgaggtcaagagat
cgagaccatcttggtcaacaaggtgaaaccccgtctctactaaaaatacaaaaattagccgggcgtggtggtgcacg
cctgtagtcccagctactcgggaggctgaggcaggagaattgcttgaacccaggaggcggaggttgcggtgagccga
gatcgtgccattgcactccagcctgggtaacAacagcgaaactccgtctc
>subfamily8 count=48 pvalue=8e-118 (parent_pvalue=8e-118) parent=1 muts=
259:g/- 190:g/a
ggccgggcgcggtggctcacgcctgtaatcccagcactttgggaggccgaggcgggtggatcacgaggtcaagagat
cgagaccatcctggccaacatggtgaaaccccgtctctactaaaaatacaaaaattagctgggcgtggtggcgcgtg
cctgtagtcccagctactcgggaggctgaggcaggagaattgcttgaacccaggaggcggaggttgcagtgagccga
gattgtgccactgcactccagcctggcgacagagcaagactctgtctc
>subfamily9 count=71 pvalue=4e-48 (parent_pvalue=8e-118) parent=1 muts=
274:t/c 174:c/a
ggccgggcgcggtggctcaagcctgtaatcccagcactttgggaggccgaggcgggtggatcacgaggtcaagagat
cgagaccatcctggtcaacatggtgaaaccccgtctctactaaaaatacaaaaattagctgggcatggtggcgcacg

cctgtagtcccagctactagggaggctgaggcaggagaattgcttgaacccaggaggcggaggttgcggtgagccga
gatcgcgccattgcactccagcctgggtaacAagagcgaaaccccgtctc
>subfamily10 count=58 pvalue=1e-26 (parent_pvalue=5e-388) parent=0 muts=
92:g/a 74:g/a
ggccgggcgcggtggctcaagcctgtaatcccagcactttgggaggccgaggcgggtggatcacgaggtcaaaagat
cgagaccatcctgatcaacatggtgaaaccccgtctctactaaaaatacaaaaaAttagctgggcatggtggcgcgt
gcctgtaatcccagctactcaggaggctgaggcaggagaattgcctgaacccaggaggcggaggttgcggtgagccg
agatcgcgccattgcactccagcctgggtaacAagagcgaaactccgtctc
>subfamily11 count=63 pvalue=6e-61 (parent_pvalue=5e-388) parent=0 muts=
171:a/- 153:g/c
ggccgggcgcggtggctcaagcctgtaatcccagcactttgggaggccgaggcgggtggatcacgaggtcaagagat
cgagaccatcctggtcaacatggtgaaaccccgtctctactaaaaatacaaaaattagctgggcgtggtggcgcctg
cctgtaatcccagctcgggaggctgaggcaggagaattgcttgaacccaggaggcggaggttgcggtgagccgagat
cgcgccattgcactccagcctgggtaacAagagcgaaactccgtctc
>subfamily12 count=55 pvalue=2e-17 (parent_pvalue=5e-388) parent=0 muts=
277:g/c 119:t/c
ggccgggcgcggtggctcaagcctgtaatcccagcactttgggaggccgaggcgggtggatcacgaggtcaagagat
cgagaccatcctggtcaacatggtgaaaccccgtctctaccaaaaatacaaaaaAttagctgggcatggtggcgcgt
gcctgtaatcccagctactcaggaggctgaggcaggagaattgcctgaacccaggaggcggaggttgcggtgagccg
agatcgcgccattgcactccagcctgggtaacAagagcgaaactccctctc
>subfamily13 count=48 pvalue=3e-48 (parent_pvalue=8e-118) parent=1 muts=
52:c/a 6:g/a
ggccggacgcggtggctcacgcctgtaatcccagcactttgggaggccgaggagggtggatcacgaggtcaagagat
cgagaccatcttggccaacatggtgaaaccccgtctctactaaaaatgcaaaaaAttagccggacgtggtggcacat
gcctgtaatcccagctacttgggaggctgagacaggagaattgcttgaacccaggaggcggaggttgcggtgagccg
agaatgcgccattgcactccagcctgggcaacaAgagcgaaactccgtctc
>subfamily14 count=40 pvalue=5e-15 (parent_pvalue=5e-388) parent=0 muts=
80:g/t 22:c/t
ggccgggcgcggtggctcaagcttgtaatcccagcactttgggaggccgaggcgggtggatcacgaggtcgagagat
ctagaccatcctggtcaacatggtgaaaccccgtctctactaaaaatacaaaaaAttagctgggcatggtggtgcgt
gcctgtaatcccagctactcaggaggctgaggcaggagaattgcctgaacccaggaggcggaggttgcggtgagccg
agatcgcgccattgcactccagcctgggtaacAagagcaaaactccgtctc
>subfamily15 count=42 pvalue=1e-17 (parent_pvalue=1e-641) parent=4 muts=
100:g/a 19:a/-
ggccgggcgcggtggctcagcctgtaatcccagcactttgggaggccgaggcgggtggatcacgaggtcgagagatc
gagaccatcctggtcaacatagtgaaaccccgtctctactaaaaatacaaaaaActagctgggcgtggtggcgcgtg
cctgtaatcccagctacttaggaggctgaggcaggagaattgcctgagcccaggaggcggaggttgcggtgagccga
gatcgcgccattgcactccagcctgggtaacGagagcgaaactccgtctc
>subfamily16 count=94 pvalue=1e-6 (parent_pvalue=5e-388) parent=0 muts=
146:g/a 98:a/t
ggccgggcgcggtggctcaagcctgtaatcccagcactttgggaggccgaggcgggtggatcacaaggtcaagagat
cgagaccatcctggtcaacatggtgaaaccccgtctctattaaaaatacaaaaaAttagctgggcatggtggggcgt
gcctgtaatcccagctactcaggaggctgaggcaggagaattgcctgaacccaggaggcggaggttgcggtgagccg
agatcgcgccattgcactccagcctgggtaacAagagcgaaactccgtctc
>subfamily17 count=49 pvalue=3e-26 (parent_pvalue=8e-118) parent=1 muts=
187:c/t 166:c/t
ggccgggcgcggtggctcacgcctataatcccagcactttgggaggccgaggcgggtggatcacgaggtcaagagat
cgagaccatcctggtcaacaaggtgaaaccccgtctctactaaaaatacaaaaattagctgggcatggtggcgcgcg
cctgtagtcctagctactcgggaggctgaggtaggagaattgcttgaactcaggaggcggaggttgcggtgagccga
gatcgtgccattgcactccagcctgggtaacAagagcgaaactccatctc
>subfamily18 count=57 pvalue=9e-26 (parent_pvalue=7e-139) parent=2 muts=
109:c/t 96:a/g
ggccgggcgcggtggctcaagcctgtaatcccagcactttgggaggccgaggcgggtggatcacgaggtcaagagat
cgagaccatcctggtcaacatggtgaaaccccgtctctactaaaaatacaaaaaaattagctgggcacgtgcctgtaa
tcccagctactcaggaggctgaggcaggagaattgcctgaacccaggaggcggaggttgcggtgagccgagatcgcg
ccattgcactccagcctgggtaacaAgagcgaaactccgtctc

>subfamily19 count=81 pvalue=2e-17 (parent_pvalue=7e-139) parent=2 muts=
109:c/t 96:a/g
ggccgggcgcggtggctcaagcctgtaatcccagcactttgggaggccgaggcgggggatcacgagggcaagagat
cgagaccatcctggtcaacatggtgaaaccccgtctctactaaaaatacaaaaattagctgggcatggtggcgcgtg
cctgtaatcccagctactcaggaggctgaggcaggagaattgcctgaacccaggaggcggaggttgcggtgagccga
gatcgcgccattgcactccagcctgggtaacAagagcgaaactccgtctc
>subfamily20 count=16 pvalue=5e-9 (parent_pvalue=4e-102) parent=3 muts=
243:t/c
ggccgggcgcggtggctcacgcctgtaatcccagcactttgggaggccgaggcgggcggatcacaaggtcaggagtt
cgagaccagcctggccaacatggtgaaaccccgtctctactaaaaatacaaaaattagctgggcgtagtggcacgca
cctgtaatcccagctactcaggaggctgaggcaggagaatcgcttgaacctgggaggcggaggttgcagtgagccga
gatcatgccactgcactccagcctgggtgacagagtgagactccgtctc
>subfamily21 count=141 pvalue=4e-174 (parent_pvalue=8e-118) parent=1 muts=
174:c/a
ggccgggcgcggtggctcacgcctgtaatcccagcactttgggaggccgaggcgggtggatcacgaggtcaagagat
cgagaccatcctggtcaacatggtgaaaccccgtctctactaaaaatacaaaaattagctgggcatggtggcgcacg
cctgtagtcccagctactagggaggctgaggcaggagaattgcttgaacccaggaggcggaggttgcggtgagccga
gatcgcgccattgcactccagcctgggtaacAagagcgaaactccgtctc
>subfamily22 count=172 pvalue=8e-49 (parent_pvalue=8e-118) parent=1 muts=
99:t/a
ggccgggcgcggtggctcacgcctgtaatcccagcactttgggaggccgaggcgggtggatcacgaggtcaagagat
cgagaccatcctggtcaacaaggtgaaaccccgtctctactaaaaatacaaaaattagctgggcatggtggcgcgtg
cctgtagtcccagctactcgggaggctgaggcaggagaattgcttgaacccaggaggcggaggttgcggtgagccga
gatcgcgccattgcactccagcctgggtaacAagagcgaaactccgtctc
>subfamily23 count=75 pvalue=2e-4 (parent_pvalue=8e-118) parent=1 muts=
243:t/c
ggccgggcgcggtggctcacgcctgtaatcccagcactttgggaggccgaggcgggtggatcacgaggtcaagagat
cgagaccatcctggtcaacatggtgaaaccccgtctctactaaaaatacaaaaattagctgggcatggtggcgcgtg
cctgtagtcccagctactcgggaggctgaggcaggagaattgcttgaacccaggaggcggaggttgcggtgagccga
gatcgcgccactgcactccagcctgggtaacAagagcgaaactccgtctc
>subfamily24 count=363 pvalue=1e-9 (parent_pvalue=8e-118) parent=1 muts=
199:t/c
ggccgggcgcggtggctcacgcctgtaatcccagcactttgggaggccgaggcgggtggatcacgaggtcaagagat
cgagaccatcctggtcaacatggtgaaaccccgtctctactaaaaatacaaaaattagctgggcatggtggcgcgtg
cctgtagtcccagctactcaggaggctgaggcaggagaattgcctgaacccaggaggcggaggttgcggtgagccga
gatcgcgccattgcactccagcctgggtaacAagagcgaaactccgtctc
>subfamily25 count=49 pvalue=1e-5 (parent_pvalue=8e-118) parent=1 muts=
212:g/a
ggccgggcgcggtggctcaagcctgtaatcccagcactttgggaggccgaggcgggtggatcacgaggtcaagagat
cgagaccatcctggtcaacatggtgaaaccccgtctctactaaaaatacaaaaattagctgggcatggtggcgcacg
cctgtagtcccagctactcgggaggctgaggcaggagaattgcttgaacccaggagacggaggttgcggtgagccga
gatcgcgccattgcactccagcctgggtaacAagagcgaaactccgtctc
>subfamily26 count=29 pvalue=5e-230 (parent_pvalue=4e-48) parent=9 muts=
210:a/g
ggccgggcgcggtggctcaagcctgtaatcccagcactttgggaggccgaggcgggtggatcacgaggtcaagagat
cgagaccatcctggtcaacatggtgaaaccccgtctctactaaaaatacaaaaattagctgggcatggtggcgcacg
cctgtagtcccagctactagggaggctgaggcaggagaattgcttgaacccagggggcggaggttgcggtgagccga
gatcgtgccattgcactccagcctgggtaacAagagcgaaaccccgtctc
>subfamily27 count=33 pvalue=2e-146 (parent_pvalue=1e-51) parent=7 muts=
96:a/g
ggccgggcgcggtggctcacgcctataatcccagcactttgggaggccgaggcgggtggatcacgaggtcaagagat
cgagaccatcttggtcagcaaggtgaaaccccgtctctactaaaaatacaaaaattagccgggcatggtggtgcacg
cctgtagtcccagctactcgggaggctgaggcaggagaattgcttgaacccaggaggcggaggttgcggtgagccga
gattgtgccattgcactccagcctgggtaacAacagcgaaactccgtctc
>subfamily28 count=48 pvalue=3e-124 (parent_pvalue=8e-118) parent=8 muts=
161:a/g

ggccgggcgcggtggctcacgcctgtaatcccagcactttgggaggccgaggcgggtggatcacgaggtcaagagat
cgagaccatcctggccaacatggtgaaaccccgtctctactaaaaatacaaaaattagccgggcatggtggcacgtg
cctgtggtcccagctactcgggaggctgaggcggaagaatcgcttgaacccaggaggcggaggttgcagtgagccga
gattgcgccactgcactccagcctggtgacagagcaagactccgtctc
>subfamily29 count=11 pvalue=2e-110 (parent_pvalue=1e-26) parent=10 muts=
254:c/a
ggccgggcgcggtggctcaagcctgtaatcccagcactttgggaggccgaggcgggtggatcacgaggtcaaaagat
cgagaccatcctgatcaacatggtgaaaccccgtctctactaaaaatacaaaaaAttagctgggcatggtggcgcgt
gcctgtaatcccagctactcaggaggctgaggcaggagaattgcctgaacccaggaggcggaggttgcggtgagccg
agatcgcgccattgcactccagactgggtaacAagagcgaaactccgtctc
>subfamily30 count=37 pvalue=2e-100 (parent_pvalue=7e-139) parent=2 muts=
148:g/t
gccgggcgcggtggctcaagcctgtaatcccagcactttgggaggccgaggcgggtggatcacaaggtcaagagatc
gagaccatcctggtcaacaaggtgaaaccccgtctctactaaaaatacaaaaaAttagctgggcatggttgtgcgtg
cctgtaatcccagctactcaggaggctgagacaggagaattgcctgaacccaggaggcggaggttgcggtgagccga
gatcgcgccattgcactccagcctgggtaacAagagcgaaactccgtctc
>subfamily31 count=20 pvalue=1e-56 (parent_pvalue=1e-51) parent=7 muts=
254:c/t
ggccgggcgcggtggctcacgcctataatcccagcactttgggaggccgaggcgggtggatcacgaggtcaagagat
cgagaccatcctggtcaacaaggtgaaaccccgtctctactaaaaatacaaaaattagctgggcatggtggtgcatg
cctgtagtcccagctactcgggaggctgaggcaggagaattgcttgaacccaggaggcggaggttgcggtgagccga
gatcgtgccattgcactccagtctgggtaacAacagcgaaactccgtctc
>subfamily32 count=20 pvalue=3e-51 (parent_pvalue=7e-139) parent=2 muts=
254:c/g
ggccgggcgcggtggctcaagcctgtaatcccagcactttgggaggccgaggcgggtggatcacaaggtcaagagat
cgagaccaacctTggtcaacaaggtgaaaccccgtctctactaaaaatacaaaaaAttagctgggcatggtggtgca
tgcctgtaatcccagctactcaggaggctgagacaggagaattgcctgaacccaggaggcggaggttgcggtgagcc
gagatcgtgccattgcactccaggctgggtaacAagagcgaaactccgtctc
>subfamily33 count=91 pvalue=3e-48 (parent_pvalue=8e-118) parent=1 muts=
88:c/t
ggccgggcgcggtggctcacgcctgtaatcccagcactttgggaggccgaggcgggtggatcacgaggtcaagagat
cgagaccattctggtcaacatggtgaaaccccgtctctactaaaaatacaaaaattagctgggcatggtggcgcacg
cctgtagtcccagctactcgggaggctgaggcaggagaattgcttgaacccaggaggcggaggttgcggtgagccga
gatcgcgccattgcactccagcctgggtaacAagagcgaaactccgtctc
>subfamily34 count=24 pvalue=4e-46 (parent_pvalue=3e-62) parent=6 muts=
171:a/t
ggccgggcgcggtggctcacgcctataatcccagcactttgggaggccgagacgggtggatcacgaggtcaagagat
cgagaccatcctggtcaacatggtgaaaccccgtctctactaaaaatacaaaaattagctgggcatggtggcgcacg
cctgtagtcccagcttctcgggaggctgaggcaggagaattgcttgaacccaggaggcggaggttgcggtgagccga
gatcgcgccattgcactccagcctgggtaacAagagcgaaactccgtctc
>subfamily35 count=28 pvalue=2e-42 (parent_pvalue=8e-118) parent=8 muts=
101:g/a
ggccgggcgcggtggctcacgcctgtaatcccagcactttgggaggccgaggcgggcggatcacgaggtcaagagat
cgagaccatcctggccatgatgaaaccccgtctctactaaaaatacaaaaattagccgggtgtggtggcacgcgcct
gtagtcccagctactcgggaggctgaggcggaagaatcgcttgaacccaggaggcggaggttgcagtgagccgagat
tgcgccactgcactccagcctggcgacagagcaagactccgtctc
>subfamily36 count=34 pvalue=3e-35 (parent_pvalue=1e-641) parent=4 muts=
204:c/a
ggccgggcgcggtggctcaagcctgtaatcccagcactttgggaggccgaggcgggtggatcacgaggtcgaaagat
cgagaccatcctggtcaacatggtgaaaccccgtctctactaaaaatacaaTAaaactagctgggcatggtggcgcg
tgcctgtaatcccagctacttaggaggctgaggcaggagaattgcctgagaccaggaggcggaggttgcggtgagcc
gagatcgtgccattgcactccagcctgggtaacAagagcgaaactccgtctc
>subfamily37 count=25 pvalue=9e-32 (parent_pvalue=1e-451) parent=5 muts=
233:g/t
ggccgggcgcggtggctcaagcctgtaatcccagcactttgggaggccgaggcgggtggatcacaaggtcgagagat
cgagaccaacctggtcaacatggtgaaaccccgtctctactaaaaatacaaaaaAttagctgggcatggtggcgcgt

gcctgtaatcccagctactcaggaggctgaggcaggagaattgcctgaacccaggaggcggaggttgcggtgagccg
atatcgcgccattgcactccagcctgggtaacAagagcgaaactccgtctc
>subfamily38 count=10 pvalue=4e-28 (parent_pvalue=8e-118) parent=8 muts=
146:g/t
ggccgggcgcggtggctcacgcctgtaatcccagcactttgggaggccgaggcaggtggatcacgaggtcaagagat
cgagaccatcctggccaacatggtgaaaccccgtctctactaaaaatacaaaaattagccgggcgtttggcacgcgc
ctgtagtcccagctactcgggaggctgaggcggaagaatcgcttgaacccaggaggcggaggttgcagtgagccgag
actgcgccactgcactccagcctggcaacagagcaagactccgtctc
>subfamily39 count=25 pvalue=4e-11 (parent_pvalue=1e-451) parent=5 muts=
174:c/g
ggccgggcgcggtggctcaagcctgtaatcccagcactttgggaggccgaggtgggtggatcacaaggtcgagagat
cgagaccaacctggtcaacatggtgaaaccccgtctctactaaaaatacaaaaaAttagctgggcatggtggcgcgt
gcctataatcccagctactgaggaggctgaggcaggagaattgcctgaacccaggaggcggaggttgcggtgagccg
agatcgcgccattgcactccagcctgggtaacAagagcgaaactccgtctc
>subfamily40 count=23 pvalue=9e-10 (parent_pvalue=8e-118) parent=1 muts=
152:c/a
ggccgggcgcggtggctcacgcctataatcccagcactttgggaggccgaggcgggtggatcacgaggtcaagagat
cgagaccatcctggtcaacatggtgaaaccccgtctctactaaaaatacaaaaattagctgggcatggtggtgagtg
cctgtagtcccagctactcgggaggctgaggcaggagaattgcttgaacccaggaggcggaggttgcggtgagccga
gatcgcgccattgcactccagcctgggtaacAagagcgaaactccgtctc
>subfamily41 count=10 pvalue=4e-7 (parent_pvalue=3e-62) parent=6 muts=
88:c/a
ggccgggcgcggtggctcacgcctataatcccagcactttgggaggccgagacgggtggatcacgaggtcaagagat
cgagaccatactggtcaacatggtgaaaccccgtctctactaaaaataaaaaaaatagctgggcatggtggcgcacg
cctgtagtcccagctactcgggaggctgaggcaggagaattgcttgaacccaggagacggaggttgcggtgagccga
gatcgcgccattgcactccagcctgggtaacagagcgaaactccgtc
>subfamily42 count=10 pvalue=4e-7 (parent_pvalue=3e-62) parent=6 muts=
108:c/g
ggccgggcgcggtggctcacgcctataatcccagcactttgggaggccgagacgggtggatcacgaggtcaagagat
cgagaccatcctggtcaacatggtgaaacgccgtctctactaaaaatacaaaaattagctgggcatggtggcgcacg
cctgtagtcccagctactcgggaggctgaggcaggagaattgcttgaacccaggagacggaggttgcggtgagccga
gatcgcgccattgcactccagcctgggtaacAagagcgaaactctgtctc
>subfamily43 count=32 pvalue=9e-6 (parent_pvalue=7e-139) parent=2 muts=
151:g/t
ggccgggcgcggtggctcaagcctgtaatcccagcactttgggaggccgaggcgggtggatcacaaggtcaagagat
cgagaccatcttggtcaacaaggtgaaaccccgtctctactaaaaatacaaaaaAttagctgggcatggtggctcgt
gcctgtaatcccagctactcaggaggctgagacaggagaattgcctgaacccaggaggcggaggttgcggtgagccg
agatcgtgccattgcactccagcctgggtaacAagagcgaaactccgtctc
>subfamily44 count=10 pvalue=3e-5 (parent_pvalue=8e-118) parent=1 muts=
212:g/c
ggccgggcgcggtggctcacgcttgtaatcccagcactttgggaggccgaggcgggtggatcacgaggtcaagagat
cgagaccatcctggtcaacatggtgaaaccccgtctctactaaaatacaaaaattagccgggcatggtggcgcacgc
ctgtagtcccagctactcaggaggctgaggcaggagaattgcttgaacccgggagccggaagttgcagtgagcagag
atcgcgccattgcactccagcctgggtaacAagagcgaaactccgtctc
>subfamily45 count=10 pvalue=1e-4 (parent_pvalue=3e-62) parent=6 muts=
173:t/c
ggccgggcgcggtggctcacgcctataatcccagcactttgggaggctgagacgggtggatcacgaggtcaagagat
cgagaccatcctggtcaacatggtgaaaccccgtctctactaaaaatacaaaaattagctgggcatggtggcacacg
cctgtagtcccagctacccgggaggctgaggcaggagaattgcttgaacccaggagacggaggttgcggtgagccga
gatcgcgccattgcactccagcctgggtaacAagagcgaaactccgtctc
