# Supplementary material for: Framework of the Alu Subfamily Evolution in the Platyrrhine Three-Family Clade of Cebidae, Callithrichidae, and Aotidae
Source: Genes (Basel). 2023 Jan 18;14(2):249. doi: 10.3390/genes14020249 (PMC9956951; doi:10.3390/genes14020249)
Supplement: Supplementary file 1 [file genes-14-00249-s001.zip › Supplementary File S4.pdf]

Alu consensus sequences for 25 Alu subfamilies lineage specific to owl monkey; Anan_2.0

>subfamily4_Owl#SINE/Alu

-GCCGGGCGCGGTGGCTCACGCCTGTAATCCCAGCACTTTGGGAGGCCGAGGCGGGTGGAT-CAC--
GAGGTCAGGAGTTCGAGACCA-GCCTGGCCAAGATGTTGAAACCCCGTCTCTACTAAAAA-TACAAAAA---
TTAGCTGGGCGTGGTGGCACGCACCTGTAATCCCAGCTACTCAGGAGGCTGAGGCAGGAGAATCGCTTGAACCTG
GGAGGCGGAGGTTGCAGTGAGCCGAGATCGCGCCATTGCACTCCAGCCTGGG------TGACA-
GAGTGAGACTCCGTCTCA-----------------------------

>subfamily22_Owl#SINE/Alu

-GCCGGGCGCGGTGGCTCACGCCTGTAATCCCAGCACTTTGGGAGGCCGAGGCGGGTGGAT-CAC--
GAGGTCAGGAGTTCGAGACCA-GCCTGACCAACATGGTGAAACCCCGTCTCTACTAAAAA-TACAAAAA---
TTAGCTGGGCGTGGTGGCACACGCCTGTAATCCCAGCTACTCAGGAGGCTGAGGCAGGAGAATTGCCTGAACCTG
GGAGGCAGAGGTTGCAGTGAGCCAAGATCGTGCCACTGCACTCCAGCCTGGG------CGACA-
GAGTGAGACTTCGTCTCA-----------------------------

>subfamily1_Owl#SINE/Alu

-GCCGGGCGCGGTGGCTCACGCCTGTAATCCCAGCACTTTGGGAGGCCGAGGCGGGCGGAT-CAC--
GAGGTCAAGAGATCGAGACCA-TCCTGGCCAACATGGTGAAACCCCGTCTCTACTAAAA-ATACAAAAA---
TTAGCCGGGCGTGGTGGCACGCGCCTGTGGTCCCAGCTACTCGGGAGGCTGAGGCAGAAGAATCGCTTGAACCC
AGGAGGCGGAGGTTGCAGTGAGCCGAGATTGCGCCACTGCACTCCAGCCTGG-------CGACA-
GAGCAAGACTCCGTCTCA-----------------------------

>subfamily21_Owl#SINE/Alu

-GCCGGGCGCGGTGGCTCACGCCTGTAATCCCAGCACTTTGGGAGGCCGAGGCGGGCGGAT-CAC--
GAGGTCAAGAGATCGAGACCA-TCCTGGCC---ATGGTGAAACCCCGTCTCTACTAAAA-ATACAAAAA---
TTAGCCGGGCGTGGTGGCACACGCCTGTGGTCCCAGCTACTCGGGAGGCTGAGGCAGAAGAATCGCTTGAACCC
AGGAGGTGGAGGTTGCAGTGAGCCGAGACTGCGCCACTGCACTCCAGCCTGG-------CAACA-
GAGCAAGACTCCGTCTCA-----------------------------

>subfamily7_Owl#SINE/Alu

GGCCGGGCGCGGTGGCTCACGCCTGTAATCCCAGCACTTTGGGAGGCCGAGGCGGGCGGAT-CAC--
GAGGTCAAGAGATCGAGACCA-TCCTCACC---ATGATGAAACCCCGTCTCTACTAAAA-
ATACAAAAAAAAATTAGCCGGGTGTGGTGGCACGCGCCTGTAGTCCCAGCTACTCGGGAGGCTGAGGCGGAAGAA
TCGCTTGAACCCAGGAGGCGGAGGTTGCAGTGAGCCGAGATTGCGCCACTGCACTCCAGCCTGG-------AGACA-
GAGCAAGACTCCGTCTCA-----------------------------

>subfamily14_Owl#SINE/Alu

---CGGGCGCGGTGGCTCACGCCTGTAATCCCAGCACTTTGGGAGGCCGAGGTGGGCGGAT-CAC--
GAGGTCAAGAGATCGAGACCA-TCCTGGCC---ATGATGAAACCCCGTCTCTACTAAAA-ATACAAAAA---
TTAGCCGGGTGTGGTGGCACGCGCCTGTAGTCCCAGCTACTCGGGAGGCTGAGGCGGAAGAATCGCTTGAACCC

AGGAGGCGGAGGTTGCAGTGAGCCGAGATTGCGCCACTGCACTCCAGCCTGG-------TGACA-
GAGCAAGACTCCGTCTCA----------------------------

>subfamily26_Owl#SINE/Alu

GGCCGGGCGCGGTGGCTCACGCCTGTAATCCCAGCACTTTGGGAGGCCGAGGCGGGCGGAT-CAC--
GAGGTCAAGAGATCGAGACCA-TCCTGGCCAACATGGTGAAACCCCGTCTCTACTAAAA-GTACAAAAAA--
TTAGCCGGGCGTGGTGGTGCGTGCCTGTAGTCCCAGCTACTCGGGAGGCTGAGGCAGGAGAATTGCTTGAAACC
AGAAAACGGAAGTTGCAGTGAGCCGGGATCGCGCCACTGCACTCCAGCCTGG-------CGATA-
GAGCAAGACTCTGTCTCA----------------------------

>subfamily13_Owl#SINE/Alu

-GCCGGGCGCGGTGGCTCACGCCTGTAATCCCAGCACTTTGGGAGGCCGAGGCGGGCGGAT-CAC--
GAGGTCAAGAGATCGAGACCA-TCCTGGCCAACATGGTGAAACCCCGTCTCTACTAAAA-ATACAAAAA---
ATAGCCGGGCATGGTGGCACGTGCCTGTGGTCCCAGCTACTCGGGAGGCTGAGGCGGAAGAATCGCTTGAACCC
AGGAGGCGGAGGTTGCAGTGAGCCGAGATTGCGCCACTGCACTCCAGCCTGG-------CGATA-
GAGCAAGACTCCGTCTCA----------------------------

>subfamily5_Owl#SINE/Alu

-GCCGGGCGCGGTGGCTCACGCCTGTAATCCCAGCACTTTGGGAGGCCGAGGCGGGCGGAT-CAC--
GAGGTCAAGAGATCGAGACCA-TCCTGGCCAACATGGTGAAACCCCGTCTCTACTAAAA-ATACAAAAA---
ATAGCCGGGCATGGTGGCACGTGCCTGTGGTCCCAGCTACTCGGGAGGCTGAGGCGGAAGAATCGCTTGAATCC
AGGAGGCGGAGGTTGCAGTGAGCCGAGATTGCGCCATTGCACTCCAGCCTGG-------CGATA-
GAGCAAGACTCCGTCTCA----------------------------

>subfamily12_Owl#SINE/Alu

-GCCGGGCGCGGTGGCTCACGCCTGTAATCCCAGCACTTTGGGAGGCCGAGGCGGGCGGAT-CAC--
GAGGTCAAGAGATCGAGACCA-TACTGGCCAACATGGTGAAACCCCGTCTCTACTAAAA-ATACAAAAA---
ATAGCCGGGCATGGTGGCACGTGCCTGTGGTCCCAGCTACTCGGGAGGCTGAGGTGGAAGAATCGCTTGAATCC
AGGAGGCGGAGGTTGCAGTGAGCCGAGATTGCGCCACTGCACTCCAGCCTGG-------CGATA-
GAGCAAGACTCCGTCTCA----------------------------

>subfamily29_Owl#SINE/Alu

-GCCGGGCGCGGTGGCTCACGCCTATAATCCCAGCACTTTGGGAGGCCGAGGCGGGTGGAT-CAC--
GAGGTCAAGAGATCGAGACCA-TCCTGGTCAACATGGTGAAACCCCGTCTCTACTAAAA-
ATACAAAAAAAATTAGCTGGGCATGGTGGCGTGCGCCTGTAGTCCCAGCTACTCGGGAGGCTGAGGCAGGAGAA
TTGCTTGAACCCAGGAGGCGGAGGTTGCGGTGAGCCGAGATCGCGCCATTGCACTCCAGCCTGGG------
TAACAAGAGCGAAACTCCGACTCA----------------------------

>subfamily11_Owl#SINE/Alu

GGCCGGGCGCGGTGGCTCACGCCTATAATCCCAGCACTTTGGGAGGCCGAGGCGGGTGGAT-CAC--
GAGGTCAAGAGATCGAGACCA-TCCTGGTCAACATGGTGAAACCCCGTCTCTACTAAAC-ATACAAAAAA--
TTAGCCGAGCATGGTGGTGCGTGCCTGTAGTCCCAGCTACTCGGGAGGCTGAGGCAGGAGAATTGCTTGAACCCA
GGAGGCGGAGGTTGCGGTGAGCCGAGATCGCGCCATTGCACTCCAGCCTGGG------
TAACAAGAGCGAAACTCCGTCTCA----------------------------

>subfamily3_Owl#SINE/Alu

-GCCGGGCGCGGTGGCTCACGCCTATAATCCCAGCACTTTGGGAGGCCGAGGCGGGTGGAT-CAC--
GAGGTCAAGAGATCGAGACCA-TCCTGGTCAACATGGTGAAACCCCGTCTCTACTAAAA-ATACAAAAA---
TTAGCTGGGCATGGTGGCGCACGCCTGTAGTCCCAGCTACTCGGGAGGCTGAGGCAGGAGAATTGCTTGAACCCA
GGAGGCGGAGGTTGCGGTGAGCCGAGATCGCGCCATTGCACTCCAGCCTGGG------
TAACAAGAGCGAAACTCCGTCTCA----------------------------

>subfamily16_Owl#SINE/Alu

-GCCGGGCGCGGTGGCTCACGCCTATAATCCCAGCACTTTGGGAGGCCGAGACGGGTGGAT-CAC--
GAGGTCAAGAGATCGAGACCA-TCCTGGTCAACATGGTGAAACCCCGTCTCTACTAAAA-ATACAAAAA---
TTAGCTGGGCATGGTGGCGCACGCCTGTAGTCCCAGCTACTCGGGAGGCTGAGGCAGGAGAATTGCTTGAACCCA
GGAGGCGGAGGTTGCGGTGAGCCGAGATCGCGCCATTGCACTCCAGCCTGGG------
TAACAAGAGCGAAACTCCGTCTCA----------------------------

>subfamily8_Owl#SINE/Alu

-GCCGGGCGCGGTGGCTCACGCCTATAATCCCAGCACTTTGGGAGGCCGAGGCGGGTGGAT-CAC--
GAGGTCAAGAGATCGAGACCA-TCCTGGTCAACAAGGTGAAACCCCGTCTCTACTAAAA-ATACAAAAA---
TTAGCTGGGCATGGTGGTGCGCGCCTGTAGTCCCAGCTACTCGGGAGGCTGAGGCAGGAGAATTGCTTGAACCCA
GGAGGCGGAGGTTGCGGTGAGCCGAGATCGCGCCATTGCACTCCAGTCTGGG------
TAACAACAGCGAAACTCCGTCTCA----------------------------

>subfamily15_Owl#SINE/Alu

-GCCGGGCGCGGTGGCTCACGCCTATAATCCCAGCACTTTGGGAGGCCGAGGCGGGTGGAT-CAC--
GAGGTCAAGAGATCGAGACCA-TCCTGGTCAACAAGGTGAAACCCCGTCTCTACTAAAA-ATACAAAAA---
TTAGCTGGGCATGGTGGCGCACGCCTGTAGTCCCAGCTACTCGGGAGGCTGAGGCAGGAGAATTGCTTGAACCCA
GGAGGCGGAGGTTGCGGTGAGCCGAGATCGCGCCATTGCACTCCAGCCTGGG------
TAACAAGAGCGAAACTCCGTCTCA----------------------------

>subfamily20_Owl#SINE/Alu

-GCCGGGCGCGGTGGCTCAAGCCTGTAATCCCAGCACTTTGGGAGGCCGAGGCGGGTGGAT-CAC--
GAGGTCAAGAGATCGAGACCA-TCCTGGTCAACATGGTGAAACCCCGTCTCTACT---A-ATACA-AAAAA-
TTAGCTGGGCATGGTGGCGCGTGCCTGTAATCCCAGCTACTCAGGAGGCTGAGGCAGGAGAATTGCCTGAACCCA
GGAGGCGGAGGTTGCGGTGAGCCGAGATCGCGCCATTGCACTCCAGCCTGGG------TA--
AAGAGCGAAACTCCGTCTCA----------------------------

>subfamily18_Owl#SINE/Alu

-GCCGGGCGCGGTGGCTCAAGCCTGTAATCCCAGCACTTTGGGAGGCCGAGGCGGGTGGAT-CAC--
GAGGTCAAGAGATCGAGACCA-TCCTGGTCAACATTGTGAAACCCCGTCTCTACT---A-ATAAA-
AAAAAATTAGCTGGGCATGGTGGCGCGTGCCTGTAATCCCAGCTACTCAGGAGGCTGAGGCAGGAGAATTGCCT
GAACCCAGGAGGCGGAGGTTGCGGTGAGCCGAGATCGCGCCATTGCACTCCAGCCTGGG------TA-
CAAGAGCGAAACTCCGTCTCA----------------------------

>subfamily10_Owl#SINE/Alu

-GCCGGGCGCGGTGGCTCACGCCTGTAATCCCAGCACTTAGGGAGGCCGAGGCGGGTGGAT-CAC--
GAGGTCAAGAGATCGAGACCA-TCCTGGTCAACATGGTGAAACCCCGTCTCTACTAAAA-ATACA-A-
AAAATTAGCTGGGCATGGTGGCGCGTGCCTGTAATCCCAGCTACTCAGGAGGCTGAGGCAGGAGAATTGCCTGA
ACCCAGGAGGCGGAGGTTGCGATGAGCCGAGATCGCGCCATTGCACTCCAGCCTGGG------
CAACAAGAGCGAAACTCCGTCTCA----------------------------

>subfamily17_Owl#SINE/Alu

-GCCGGGCGCGGTGGCTCAAGCCTGTAATCCCAGCACTTTGGGAGGCCGAGGCGGGTGGAT-CAC--
GAGGTCAAGAGATCGAGACCA-TCCTGGTCAACATTGTGAAACCCCGTCTCTACT---A-ATACA-
AAAAAATTAGCTGGGCATGGTGGCGCGTGCCTGTAATCCCAGCTACTCAGGAGGCTGAGGCAGGAGAATTGCCT
GAACCCAGGAGGCGGAGGTTGCGGTGAGCCGAGATCGCGCCATTGCACTCCAGCCTGGG------
TAACGAGAGCGAAACTCCGTCTCA----------------------------

>subfamily23_Owl#SINE/Alu

-GCCGGGCGCGGTGGCTCAAGCCTGTAATCCCAGCACTTTGGGAGGCCGAGGCGGGTGGAT-CAC--
GAGGTCAAGAGATCGAGACCA-TCCTGGTCAACATGGTGAAACCCCGTCTCTACT---A-ATACA-AAAAA-
TTAGCTGGGCATGGTGGCGCGTGCCTGTAATCCCAGCTACCCAGGAGGCTGAGGCAGGAGAATTGCCTGAACCCA
GGAGGCGGAGGTTGCGGTGAGCCGAGATCGCGCCATTGCACTCCAGCCTGGG------
TAACGAGAGCGAAACTCCGTCTCA----------------------------

>subfamily25_Owl#SINE/Alu

-GCCGGGCGCGGTGGCTCAAGCCTGTAATCCCAGCACTTTGGGAGGCCAAGGCGGGTGGAT-CAC--
GAGGTCAAGAGATCGAGACCA-TCCTGGTAAACATGGTGAAACCCCGTCTCTACT---A-ATACA-AAAAA-
TTAGCTGGGCATGGTGGCGCGTGCCTGTAATCGCAGCTACTCAGGAGGCTGAGGCAGGAGAATTGCCTGAACCC
AGGAGGCGGAGGTTGCGGTGAGCCGAGATCGCGCCATTGCACTCCAGCCTGGG------
TAACAAGAGCGAAACTCCGTCTCA----------------------------

>subfamily24_Owl#SINE/Alu

GGCCGGGCGCGGTGGCTCAAGCCTGTAATCCCAGCACTTTGGGAGGCCGAGGCGGGTGGAT-CAC--
GAGGTCAAGAGATCGAGACCA-TCCTGGTCAACATGGTGAAACCCCGTCTCTACTAA----TACA-A-
AAAATTAGCTGGGCACGGTGGCGCGTGCCTGTAATCCCAGCTACTCAGGAGGCTGAGGCAGGAGAATTGCCTGA
ACCCAGGAGGCGGAGATTGCGGTGAGCCGAGATCGCGCCATTGCGCTCCAGCCTGG-------
TATCAAGAGCGAAACTCCGTCTCA----------------------------

>subfamily19_Owl#SINE/Alu

-GCCGGGCGCGGTGGCTCAAGCCTGTAATCCCAGCACTTTGGGAGGCCGAGGCGGGTGGAT-CAC--
GAGGTCGAGAGATCGAGACCA-TCCTGGTCAACATTGTGAAACCCCGTCTCTACT---A-ATACA-
AAAAAATTAGCTGGGCACGGTGGCGCGTGCCTGTAATCCCAGCTACTCAGGAGGCTGAGGCAGGAGAATTGCCT
GAACCCAGGAGGCGGAGGTTGCGGTGAGCCGAGATCGCGCCATTGCACTCCAGCCTGGG------
TAACAAGAGCGAGACTCCGTCTCA----------------------------

>subfamily9_Owl#SINE/Alu

GGCCGGGCGCGGTGGCTCAAGCCTGTAATCCCAGCACTTTGGGAGGCCGAGGCGGGTGGAT-CAC--
GAGGTCGAGAGATCGAGACCA-TCCTGGTCAACATTGTGAAACCCCGTCTCTATT---A-ATACA-

AAAAAATTAGCTGGGCACGGTGGCGCGTGCCTGTAATCCCAGCTACTCAGGAGGCTGAGGCAGGAGAATTGCCT
GAACCCAGGAGGCGGAGGTTGCGGTGAGCCGAGATCGCGCCATTGCACTCCAGCCTGGG------
TAACAAGAGCGAGACTCCGTCTCA-----------------------------
