# Supplementary material for: Framework of the Alu Subfamily Evolution in the Platyrrhine Three-Family Clade of Cebidae, Callithrichidae, and Aotidae
Source: Genes (Basel). 2023 Jan 18;14(2):249. doi: 10.3390/genes14020249 (PMC9956951; doi:10.3390/genes14020249)
Supplement: Supplementary file 1 [file genes-14-00249-s001.zip › Supplementary File S5.pdf]

Alu consensus sequence for 8 Alu subfamilies lineage specific to Cebus monkey [Cebus_imitator-1.0]

>subfamily1_Ceb

-GCCGGGCGCGGTGGCTCACGCCTGTAATCCCAGCACTTTGGGAGGCCGAGGCGGGTGGAT-CAC--
GAGGTCAAGAGATCGAGACCA-TCCTGGCCAACATGGTGAAACCCCGTCTCTACTAAAA-ATACAAAAA---
TTAGCTGGGCATGGTGGCACGCGCCTGTAGTCCCAGCTACTCGGGAGGCTGAGGCAGGAGAATTGCTTGAACCCA
GGAGGCGGAGGTTGCAGTGAGCCGAGATCGCGCCACTGCACTCCAGCCTGG-------CGACA-
GAGCGAGACTCCGTCTCA----------------------------

>subfamily6_Ceb

GGCCGGGCGCGGTGGCTCACGCCTGTAATCCCAGCACTTTGGGAGGCCGAGGCGGGCGGAT-CAC--
GAGGTCAAGAGATCGAGACCA-TTCTCGCC---ATGATGAAACCCCGTCTCTACTAAAA-ATACAAAAA---
TTAGCCGGGTGTGGTGGCACGCGCCTGTAGTCCCAGCTACTCGGGAGGCTGAGGCGGAAGAATCGCTTGAACCC
AGGAGGCGGAGGTTGCAGTGAGCCGAGATTGCGCCACTGCACTCCAGCCTGG-------CGACA-
GAGCAAGACTCCGTCTCA----------------------------

>subfamily7_Ceb

-GCCGGGCGCGGTGGCTCACGCCTGTAATCCCAGCACTTTGGGAGGCCGAGGCGGGCGGAT-CAC--
GAGGTCAAGAGATCGAGACCA-TCCTGGCCAACATGGTGAAACCCCGTCTCTACTAAAA-ATACAAAAA---
TTAGCCGGGCATGGTGGCACGCGCCTGTGGTCCCAGCTACTCGGGAGGCTGAGGCGGAAGAATCGCTTGAACCC
AGGAGGCGGAGGTTGCAGTGAGCCGAGATTGCGCCACTGCACTCCAGCCTGG-------TGACA-
GAGCAAGACTCCGTCTCA----------------------------

>subfamily3_Ceb

-GCCGGGCGCGGTGGCTCACGCCTATAATCCCAGCACTTTGGGAGGCCGAGGCGGGTGGAT-CAC--
GAGGTCAAGAGATCGAGACCA-TCCTGGTCAACAAGGTGAAACCTCGTCTCTACTAAAA-ATACAAAAA---
TTAGCTGGGCATGATGGTGCGTGCCTGTAGTCCCAGCTACTCGGGAGGCTGAGGCAGGAGAATTGCTTGAACCCA
GGAGGCGGAGGTTGCGGTGAGCCGAGATCGTGCCATTGCACTCCAGTCTGGG------
CAACAACAGCGAAACTCCGTCTCA----------------------------

>subfamily5_Ceb

-GCCGGGCGCGGTGGCTCAAGCCTGTAATCCCAGCACTTTGGGAGGCCGAGGCGGGTGGAT-CAC--
GAGGTCAAGAGATCGAGACCA-TCCTGGTCAACAAGGTGAAACCTCGTCTCTACTAAAA-ATACAAAAA---
TTAGCTGGGCATGATGGTGCATGCCTGTAGTCCCAGCTACTCGGGAGGCTGAGGCCGGAGAATTGCTTGAACCCA
GGAGGCGGAGGTTGCGGTGAGCCGAGATCGTGCCATTGCACTCCAGCCTGGG------
CAACAACAGCAAAACTCCGTCTCA----------------------------

>subfamily4_Ceb

-GCCGGGCGCGGTGGCTCACGCCTGTAATCCCAGCACTTTGGGAGGCCGAGGCGGGTGGAT-CAC--
GAGGTCAAGAGATCGAGACCA-TCCTGGTCAAAATGGTGAAACCCCGTCTCTACTAAAA-ATACA-A-
AAAATTAGCTGGGCATGGTGGCGCGTGCCTGTAATCCCAGCTACTCAGGAGGCTGAGGCAGGAGAATTGCCTGA

ACCCAGGAGGCGGAGGTTGCGGTGAGCCGAGATCGCGCCATTGCACTCCAGCCTGGG------
TAACAAGAGCGAAACTCCGACTCA----------------------------

>subfamily2_Ceb

-GCCGGGCGCGGTGGCTCAAGCCTGTAATCCCAGCACTGTGGGAGGCCGAGGCGGGTGGAT-CAC--
GAGGTCAAGAGATCGAGACCA-TCCTGGTCAACATGGTGAAACCCCGTCTCTACTAAAA-ATACA-AAAAA-
TTAGCTGGGCATGGTGGCGCGTGCCTGTAATCCCAGCGACTCAGGAGGCTGGGGCAGGAGAATTGCCTGAACCC
GGGAGGCGGAGGTTGCGGTGAGCCGAGATCGCGCCATTGCACTCCAGCCTGGG------
TAACAAGAGCGAAACTCCGTCTCA----------------------------

>subfamily8_Ceb

-GCCGGGCGCGGTGGCTCAAGCCTGTAATCCCAGCACTGTGGGAGGCCGAGGCGGGTGGAT-CAC--
GAGGTCAAGAGATCGAGACCA-TCCTGGTCAACATGGTGAAACCCCGTCTCTACTAAAA-ATACA-AAAAA-
TTAGCTGGGCATGGTGGCGCGTGCCTGTAATCCCAGCGACTCAGGAGGCTGGGGCAGGAGAATTGCCTGAACCC
GGGAGGCGGAGGTTGCGGTGAGCCGAGATCGCGCCATTGCACTCCAGCCTGGG------
TAACAAGAGGGAAACTCCGTCTCA----------------------------
